# Perceived fairness of direct-to-consumer genetic testing business models

**DOI:** 10.1007/s12525-022-00571-x

**Published:** 2022-07-18

**Authors:** Philipp A. Toussaint, Scott Thiebes, Manuel Schmidt-Kraepelin, Ali Sunyaev

**Affiliations:** Department of Economics and Management, Karlsruhe Institute of Technology, Kaiserstr. 89, 76133 Karlsruhe, Germany

**Keywords:** Direct-to-consumer genetic testing, Business models, Retail fairness, Genetic privacy, Discrete choice experiment, D12, D22, D49, I11

## Abstract

Although consumers and experts often express concerns regarding the questionable business practices of direct-to-consumer (DTC) genetic testing services (e.g., reselling of consumers’ genetic data), the DTC genetic testing market keeps expanding rapidly. We employ retail fairness as our theoretical lens to address this seeming paradox and conduct a discrete choice experiment with 16 attributes to better understand consumers’ fairness perceptions of DTC genetic testing business models. Our results suggest that, while consumers perceive privacy-preserving DTC genetic testing services fairer, price is the main driver for fairness perception. We contribute to research on consumer perceptions of DTC genetic testing by investigating consumer preferences of DTC genetic testing business models and respective attributes. Further, this research contributes to knowledge about disruptive business models in healthcare and retail fairness by contextualizing the concept of retail fairness in the DTC genetic testing market. We also demonstrate how to utilize discrete choice experiments to elicit perceived fairness.

**Supplementary Information:**

The online version contains supplementary material available at 10.1007/s12525-022-00571-x.

## Introduction

Driven by the dwindling costs for collecting and analyzing genetic data, numerous direct-to-consumer (DTC) genetic testing services emerged in the mid-2000s (Allyse et al., 2018). Increasing public interest in genetics and genetic testing has led to a rapid expansion of the DTC genetic testing market (Ramos & Weissman, 2018), which had an estimated value of $1.09 billion in 2019, and is estimated to triple by 2028 (Ugalmugle & Swain, 2020). Unsurprisingly, hundreds of companies worldwide nowadays offer genetic testing directly to consumers (Phillips, 2018).

With DTC genetic testing being a relatively young and disruptive business sector (Turrini, 2018), the DTC genetic testing market is still largely unregulated. Paired with genetic data being among the most sensitive personal data, this has led to several questionable business practices by DTC genetic testing service providers (Ramos & Weissman, 2018). Due to its market novelty and overall lack of governmental oversight (Allyse et al., 2018), critics argue against service providers’ unregulated advertising and marketing claims, lack of clinical test validity, lack of meaningful test result interpretation, use of collected genetic data for undisclosed research purposes, or reselling of genetic data to third parties (Haga & Willard, 2008; Hudson et al., 2007; Hunter et al., 2008; Ramos & Weissman, 2018; Thiebes et al., 2020). For example, 23andMe, one of the largest DTC genetic testing services providers, is known to resell its customers’ genetic data to clinical research and biopharmaceutical companies (Raz et al., 2020). Toward that end, 23andMe announced in 2018 that British pharma firm GlaxoSmithKline invested $300 million into the company to access its genetics research database (Hamzelou, 2020). By comparison, 23andMe reported a total revenue of approximately $260 million for 2021 (Schafer, 2021). Experts have repeatedly voiced their own and consumers’ concerns about service providers utilizing such practices (Briscoe et al., 2020; Majumder et al., 2016). Extant research also shows that consumers’ concerns regarding DTC genetic testing business practices are not limited to reselling genetic data to pharmaceutical companies (Ramos & Weissman, 2018), but encompass multiple aspects of genetic privacy, such as access to genetic data by insurance companies, employers, law enforcement, or malicious entities like hackers (Majumder et al., 2021). Given the numerous times that concerns have been raised over DTC genetic testing services business practices, one would expect that such practices are perceived as unfair, resulting in adverse effects on consumer adoption. Yet, in practice, DTC genetic testing is gaining popularity among consumers with an ever-increasing demand for genetic testing services (Ramos & Weissman, 2018; Raz et al., 2020).

The seeming paradox that, on the one hand, consumers are concerned about certain DTC genetic testing business practices but, on the other hand, increasingly engage with DTC genetic testing despite these concerns is opposed by research and practice in other disruptive markets. For example, regarding IT services and e-commerce, it has been demonstrated that consumer fairness is an important factor for long-term customer satisfaction and market success (Carr, 2007; Nguyen & Klaus, 2013). Because DTC genetic testing business models are diverse and ever-evolving, they can be very complex and often consist of many different attributes. Despite sharing many similar attributes to other markets, these business models also entail distinct features, such as genetic test type, storage of the raw DNA sample as well as processed genetic data, and possible reselling of genetic data (Thiebes et al., 2020). Hence, investigating the perceived fairness of extant DTC genetic testing business model attributes could help shed light on the seeming paradox of how the DTC genetic testing market is growing rapidly while many consumers and experts are concerned regarding their genetic privacy. Moreover, it can help us understand whether specific attributes outweigh consumers’ concerns and unfair perceptions of other attributes. Therefore, we ask the following research question:

- **RQ:** How do observable attributes of DTC genetic testing business models influence consumers’ perceived fairness of these business models?

Within the healthcare sector, the digital transformation of business models is often discussed in the literature (e.g., Gleiss et al., 2021; Hwang, 2008). Moreover, research has also investigated fairness in digital healthcare and nascent e-commerce sectors (e.g., Constantiou et al., 2012; Han et al., 2008). Regarding DTC genetic testing, extant research on fairness has mainly investigated fairness in the context of laws and regulations (e.g., de Vries et al., 2015) or terms of services (e.g., Phillips, 2017). However, research analyzing consumers’ perceived fairness of DTC genetic testing or their respective business models is still scarce. Toward this end, we lack knowledge on how certain business practices shape consumers’ perceptions of fairness in DTC genetic testing. To better understand consumer fairness in DTC genetic testing, we design and conduct a discrete choice experiment (DCE) to elicit consumers’ fairness perceptions of DTC genetic testing business models. For this, we draw on retail fairness as a theoretical foundation for fairness (Nguyen & Klaus, 2013).

With this study, we contribute to research and practice on several levels. For research, we add to the literature on consumer perceptions of DTC genetic testing by investigating consumer preference for DTC genetic testing business models and respective attributes. We also contribute to the research streams of disruptive business models in healthcare and retail fairness by contextualizing the concept of retail fairness in the DTC genetic testing market. We further contribute to the literature by demonstrating how DCEs can be used to elicit perceived fairness. For practice, our choice model may be a valuable tool for DTC genetic testing service providers to assess consumers’ perceived fairness of their business model and compare it with other business models. Moreover, our findings could aid policymakers in creating fair and informed regulations for the DTC genetic testing market, protecting consumers’ interests while ensuring a free market economy.

The remainder of this paper is structured as follows. Next, we provide a detailed introduction to DTC genetic testing and retail fairness. In section three, we outline our four-stage research approach for designing and conducting the DCE and present the results of our experiment in section four. Finally, we discuss the results of our DCE in section five and end with a brief conclusion in section six.

## Related work

### Direct-to-consumer genetic testing business models

In contrast to traditional clinical genetic testing, the consumers initiate DTC genetic tests with no need for personal interactions with healthcare professionals (Ramos & Weissman, 2018). Typically, DTC genetic tests are advertised and sold to consumers via the Internet. Upon purchase, the service provider sends a DNA sample collection kit (e.g., buccal swab or blood-spot collection) to the consumer for self-collection or arranges for a sample collection at a local laboratory (Thiebes et al., 2020). Once the sample is retrieved, the service provider performs the genetic test and returns the results to the consumer, usually via the Internet or by mail (Hudson et al., 2007). This process means that consumers choose the interpreter of their genetic information, as opposed to the healthcare provider interpreting the genetic data for the consumer, and that consumers are responsible for managing and ensuring the privacy and security of their genetic data (Allyse et al., 2018). The most common types of DTC genetic testing services on the market include lifestyle tests (e.g., ancestry, traits, and nutrition), relationship tests (e.g., paternity), and medical health tests (Ramos & Weissman, 2018).

Although in the US, DTC medical tests require clearance by the US Food and Drug Administration, supporters of traditional clinical genetic testing frequently criticize the quality and clinical validity of these tests and the interpretability of test results by consumers. They state that the results are often misleading and should be overseen by healthcare professionals (Allyse et al., 2018). Another major concern is the handling of genetic data. DTC companies often build large databases of their customers’ data and utilize this data for product research or revenue increase by selling it to clinical research or biopharmaceutical companies (Allyse et al., 2018; Raz et al., 2020). Hence, numerous studies have investigated genetic privacy and sharing of genetic data from ethical (e.g., Lewis et al., 2013; Riso et al., 2017), legal (e.g., Ducournau et al., 2013; Hogarth et al., 2008; Hudson et al., 2007), and social sciences (e.g., Anderson & Agarwal, 2009, 2011; Thiebes et al., 2017) standpoints. While the DTC genetic testing market is ever-growing (Ugalmugle & Swain, 2020), controversy about service providers’ business practices and genetic privacy continues. With consumers’ needs for ethicality, privacy, and better treatments rising (Critchley et al., 2017), genetic privacy and sharing of personal data remain a subject of scholarly debate (Hendricks-Sturrup & Lu, 2019).

Within a growing market and evolving regulations, DTC genetic testing service providers have adopted many different business models. Drawing on Shafer et al. (2005) and aligning our view with Thiebes et al. (2020), we understand a business model as consisting of four major categories of components: (1) strategic choices (e.g., customers, target markets, value propositions, revenues and pricing, competitors), (2) value creation (e.g., key resources, assets, processes), (3) value network (e.g., information and product flows between an organization, its suppliers, and customers), and (4) capturing value (e.g., profit-making mechanisms). While extant research has investigated business models in the healthcare market (e.g., Gleiss et al., 2021; Hwang, 2008), research on business and marketing aspects of DTC genetic testing is still scarce. Literature closest to this study engages with socioeconomic aspects such as research on how marketing strategies of DTC genetic testing services impact consumers (e.g., Ducournau et al., 2013), the impact of consumers’ genetic variations on their economic behaviors (e.g., Cesarini et al., 2012; Daviet et al., 2021; Kock, 2009), socioeconomic implications of consumers sharing their genetic data freely (e.g., Riso et al., 2017; Vassilakopoulou et al., 2019), or digital entrepreneurs appropriating value from genetic data (e.g., Jarvenpaa & Markus, 2018; Rothe et al., 2019). A first overview of DTC genetic testing service business models is provided by Thiebes et al. (2020), who analyzed the business models of 277 DTC genetic testing services and developed a comprehensive taxonomy of business models in DTC genetic testing, which consists of 15 dimensions and 41 characteristics and is organized along the four major business model categories introduced above (i.e., strategic choices, value network, create value, and capturing value). Based on their taxonomy, they additionally derived six prevalent archetypes of DTC genetic testing business models, namely (1) low-cost DTC genomics for enthusiasts, (2) high-privacy DTC genomics for enthusiasts, (3) specific information tests, (4) simple health tests, (5) basic low-value DTC genomics, and (6) comprehensive tests and low data processing.

Although these studies provide valuable first insights into the landscape of DTC genetic testing business models that can serve as a basis for our empirical inquiry, consumer perceptions of business practices and business models in DTC genetic testing remain understudied and require further investigation.

### Retail fairness

The study of perceived fairness in psychology, marketing, information systems, and related research areas can be traced back to John Stacie Adam’s seminal work on equity theory within social exchanges (Adams, 1963, 1965). Originally, equity theory solely focused on *distributed justice*, which refers to a phenomenon where individuals assess the fairness of an exchange by comparing their inputs to the outcomes they receive and thereby calculate an equity score (Maxham & Netemeyer, 2002). It should be noted that the terms justice and fairness are often used interchangeably in the business and marketing context (Seiders & Berry, 1998). When observing distributive justice, an exchange is typically perceived as fair by an individual when their equity score is proportional to the scores of referent others, such as similar customers (Deutsch, 2010; Maxham & Netemeyer, 2002). Following the inability of equity theory and other models of distributive justice (e.g., Crosby, 1976; Deutsch, 2010) to explain and predict individuals’ reactions to perceived injustice, research expanded its conceptualization toward *procedural justice*, which refers to the perceived fairness of the process by which outcomes are determined (Cohen-Charash & Spector, 2001). Studies in this area showed that the distribution of rewards (i.e., distributive justice) is sometimes less relevant to determining perceived fairness than the process itself by which rewards were allocated (Cohen et al., 1989). Lastly, *interactional justice* represents an extension of procedural justice that emphasizes the influence of the interpersonal treatment that customers or employees experience during a process of exchange (Cohen-Charash & Spector, 2001). In particular, interactional justice describes the perception of the communication process between a justice source (e.g., company representative) and a justice recipient (e.g., customer), such as politeness, honesty, and respect (Bies, 1986).

Since then, numerous studies have demonstrated that perceived fairness is an important, multidimensional concept for customer satisfaction in various settings, ranging from traditional offline businesses like banking or hospitality to modern businesses like e-commerce and IT service (e.g., Carr, 2007; Constantiou et al., 20122; Han et al., 2008; Zhu & Chen, 2012). Furthermore, investigations of fairness are rapidly gaining importance and require new perspectives as companies more and more shift toward Internet-based business models. This is due to two significant differences between the offline and the e-commerce world. First, traditional commerce requires personal interaction, and as such, the fairness perception is influenced mainly by interpersonal interactions. In contrast, on the Internet, business-customer relations are bound to the service provider’s website (i.e., interactional justice may become less relevant). Second, from a customer’s perspective, evaluating the fairness of an offered good or service is quicker online as the comparison of service providers and exchange of opinions with other customers is swifter on the Internet due to search engines and discussion boards. Although this feature of Internet-based business models, at first glance, facilitates the necessary process of comparing outcomes as described by equity theory (Adams, 1963, 1965), it also implies new problems, like not physically witnessing other customers and their interactions with the service provider (Zhu & Chen, 2012). Numerous studies conducted on pre-Internet-supported business models have employed service fairness to measure and describe fairness (e.g., Namkung et al., 2009). For this, service fairness usually relies on a negative event the customer experiences, resulting in the perception of unfair treatment (Mccoll-Kennedy & Sparks, 2003). Current technology and the shift to e-commerce allow for increased use of business practices that may be considered unfair (e.g., tracking devices such as RFID tags, face recognition, website tracking, or reselling genetic data) (Nguyen & Klaus, 2013; Raz et al., 2020). Therefore, while previous research focuses on fairness in the context of service failure and recovery (e.g., Mccoll-Kennedy & Sparks, 2003) or in specific settings such as the hospitality industry (e.g., Namkung et al., 2009), Nguyen and Klaus (2013) argue that fairness should additionally expand on honesty, integrity, transparency, and ethical behavior of service providers. To address these shortcomings, they adopt a holistic view of fairness and develop a conceptual framework for general *retail fairness* with the three dimensions *product*, *interaction*, and *service*.

While we understand DTC genetic testing as a service, the utilized business model taxonomy by Thiebes et al. (2020) primarily describes business-customer relations concerning the unique attributes of DTC genetic testing, all of which are *product*, *interaction*, and *service* aspects of retail. Aligning our view with Nguyen and Klaus (2013), we define fairness as “the degree of perceived quality, honesty, and justice a customer has for a retailer.“ To date, research on fairness in DTC genetic testing is limited. Extant literature has addressed fairness in the context of laws and regulations for genetic testing or genetic data (e.g., de Vries et al., 2015; Morrow, 2009), security of research participants donating genetic data (e.g., Newcomb, 2010), or terms of services of DTC genetic testing providers (e.g., Phillips, 2017). However, we still lack knowledge on the fairness of DTC genetic testing business models. Furthermore, the related research stream on consumer preference in DTC genetic testing is also scarce. Most published research focuses on the preference for preemptive clinical genetic testing for diseases (e.g., Blumenschein et al., 2016; Najafzadeh et al., 2013), preference for prioritizing different clinical genetic tests (e.g., Severin et al., 2015), or willingness to pay (WTP) for clinical genetic testing (e.g., Dong et al., 2016). Consequently, our literature review resulted in only one study investigating consumer preference for DTC genetic testing. Jeong (2017) conduct a DCE on consumer preference toward DTC genetic testing products (price, testable items, test accuracy, and possibility of information leaks), assessing the 2016 Korean DTC genetic testing policies and regulations. Their research shows that consumers prefer a DTC genetic test that is cheap, tests various items or genes, offers accurate test results, and guarantees the confidentiality of all information.

## Methods

**Fig. 1:**
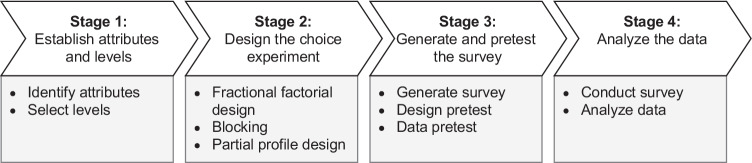
Overview of the research approach

To explore the perceived fairness of DTC genetic testing business models, we conducted a DCE among potential DTC genetic testing consumers. Rooted in marketing, DCEs provide insights into consumer preference toward products and services not (yet) available on the market. This is achieved by asking individuals to state their preferred choice from different hypothetical alternatives, allowing researchers to reveal how individuals value selected attributes of a product or service (Mangham et al., 2009). DCEs can be used for a variety of preference variables, like preference to buy a product and WTP (Meenakshi et al., 2012), perceived attribute importance (Sculpher et al., 2004), or preference for potential benefits from switching service providers (Ryan et al., 2001). Since the DTC genetic testing market is constantly evolving and consumer preferences are mostly unknown, a DCE allows us to compare hypothetical DTC genetic testing services and elicit consumers’ perceptions of specific business model attributes. In particular, consumer fairness perception can be investigated independently of existing services focusing on the business model attributes and their possible manifestations. Hereafter we explain the four stages of conducting our DCE in more detail. Figure 1 provides an overview of the different steps for each stage of the DCE.

### Establishing attributes and assigning levels

The first stage of a DCE is to identify the attributes relevant to the stated research question, which are then filled with different levels (Mangham et al., 2009). An attribute may be any characteristic describing the good or service, while the different levels are manifestations of each attribute (Ryan et al., 2001). Because the hypothetical scenarios are constructed utilizing the attributes and levels, it is crucial to select the right set of attributes for the given research question. Following Lancsar and Louviere (2008), we drew on select literature, namely, the taxonomy of business models in DTC genetic testing from Thiebes et al. (2020), to deduce the attributes and levels. Since the taxonomy was constructed by analyzing existing services, it provided us with attributes and levels reflecting real DTC genetic testing business models. Nonetheless, this also allows for the construction of new hypothetical business models through the targeted combination of levels. Therefore, as a first step, we defined all 15 dimensions as attributes with the 41 characteristics of the taxonomy as their respective levels. Next, we inspected our newly found attributes regarding the relevance for perceived fairness of DTC genetic testing consumers. This led to the removal of the *consumer target group*, as this is not an attribute that can change with the business model but rather describes the consumer. Instead, we added a new attribute named *test purpose* reflecting the three main genetic testing categories *lifestyle tests, relationship tests*, and *health tests*. These levels largely depict the three consumer target groups of enthusiasts, specific information seekers, and chronic health issue & risk groups and provide respondents with a more fine-grained understanding of what the hypothetical service is offering. Additionally, we removed the *fee type* dimension. For this, we replaced it with the *price* attribute, with different prices as levels and another *additional value subscription* attribute. These changes were made for two reasons. First, a concrete price is closer to an actual market situation and allows respondents to better evaluate hypothetical business models (Hall et al., 2004). Second, including price as a numerical attribute enables WTP calculation (a monetary measure of benefits) (Ryan et al., 2001). The use of a cost or price attribute is very common in consumer research, especially DCEs, as it represents the main trade-off to the features a product may have; in other words, the price is what the consumer needs to give to receive the product (Hall et al., 2004). Further, we undertook some minor attribute and level naming changes. Concerning the choice of levels, there is no limit on the number of levels an attribute can have. Nonetheless, McCullough (2002) recommends limiting the number of levels per attribute to a realistic minimum. Also, one should keep the number of levels across all attributes as even as possible to minimize the number of levels effect, where an attribute with a higher number of levels becomes more important than an attribute with fewer levels (McCullough, 2002). Since the 16 attributes of this study consist of two or three levels each, these recommendations are met. An overview of the final selection of attributes and their respective levels is shown in Table 1. For a detailed description of all attributes and levels, refer to Supplementary Material 1.

**Table 1:** Attributes and levels of DTC genetic testing business models

Attribute	Level 1	Level 2	Level 3
Test purpose	Health test	Lifestyle test	Relationship test
Business purpose	For-profit	Nonprofit	-
Region of operation	Local	Worldwide	-
Consumer research consent	Data not used	Mandatory	Optional
Distribution channel	Healthcare professionals only	Internet only	Multi-contact service
Sampling site	Home collection	Lab collection	Home or Lab collection
Sampling kit provider	Service provider	Third party	Service provider or Third party
Sample storage	Consumer decision	Mandatory	Never
Genome test type	Genotyping	Sequencing	Genotyping or Sequencing
Data storage	No storage	Isolated storage	Database for service provider
Data ownership	Consumer	Service provider	-
Data processing	No interpretation	Basic interpretation	Value-added interpretation
Price	$0	$100	$1000
Additional value subscription	No	Yes	-
Partial coverage by insurance	No	Yes	-
Reselling of genome data	No	Yes	-

### Designing the choice experiment

The second stage of a DCE concerns the actual design of the choice experiment. For this, hypothetical alternatives need to be created, which are then combined into different choice sets (Mangham et al., 2009). Due to a large number of attributes in this study, we implemented a fractional factorial design with blocking and partial profiles. To begin with, we used SAS OnDemand to determine the number of choice sets needed to create an efficient fractional factorial design with our 16 attributes and 42 levels. The fractional factorial design holds a subset of the full factorial design such that all main effects and as many higher-order interactions as possible are still estimable (Lancsar & Louviere, 2008).

For this, we utilized the %*MktRuns* Macro, which finds design sizes, where balance and orthogonality are perfect or near-perfect (i.e., optimal D-efficiency) (Kuhfeld, 2010; Mangham et al., 2009). The *%MktRuns* showed that a saturated design could be made with as little as 27 choice sets, with the smallest perfectly efficient design needing a minimum of 36 choice sets. Previous literature has shown that respondents can answer 20 or more choice sets without degradation of data quality (Lancsar & Louviere, 2008; McCullough, 2002), however as a precaution and to prevent boredom, it is recommended to limit the tasks to about 10–15, including warmup tasks (Mangham et al., 2009; McCullough, 2002). In contrast, larger designs provide more statistical information and minimize bias (Lancsar & Louviere, 2006). Therefore, we decided to use 144 choice sets but split them into 12 blocks with 12 choice sets each, leaving enough headroom for the profiling process. Blocking reduces the workload of a single participant by efficiently splitting the design into different versions for multiple respondents (Lancsar & Louviere, 2008). Knowing that we wanted a partial profile design to reduce the number of attributes respondents would have to compare at a time, we used the *%MktBSize* Macro to determine the number of attributes that vary for each choice set. While the Macro is primarily used to find sizes for block designs, it can also be used for the above task. Executing the *%MktBSize* Macro with 16 attributes yielded the viable solutions with *k* = 6 or *k* = 10 attributes varying per choice set (i.e., six attributes may vary their levels while ten have the same levels or ten attributes may vary their levels while six are held constant). Since both solutions only require eight choice sets per respondent, we chose the smaller option with six varying attributes. The last important decision concerning the design was selecting the number of hypothetical alternatives each choice set should contain. While there is no upper limit, respondents need to be able to decide between the offered alternatives reasonably. In practice, most DCEs include two to five alternatives (McCullough, 2002). Additionally, many experiments include a non-choice option, allowing respondents to choose neither option if all alternatives are unappealing (Lancsar & Louviere, 2008). This also more closely resembles a real-world context, as individuals are not always required to make a choice (Mangham et al., 2009). Because we had a relatively large number of attributes (although only six vary at a time), we decided to display three alternatives and a non-choice option (i.e., four options) per choice set. With all design decisions in place, we utilized JMP Version 15.1.0 to create a D-efficient choice design for our 144 choice sets in 12 blocks.

### Generating and pretesting the survey

**Fig. 2:**
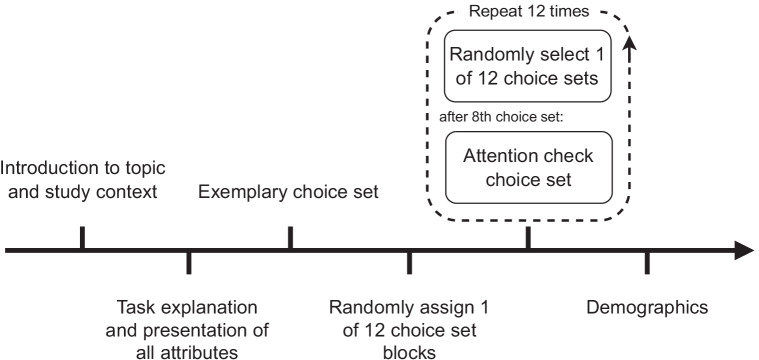
Overview of the survey procedure

After the choice sets were generated, the third stage involved collecting respondent data. This required determining the inclusion/exclusion criteria of respondents as well as the number of participants (i.e., sample size) (Lancsar & Louviere, 2008). Because it is important to include existing consumers, potential consumers, and also non-potential consumers in DCEs to investigate the potential acceptance of a new (hypothetical) product, it is also important to include all consumer groups when analyzing fairness perceptions of business models. Since every (legal aged) citizen can be a potential consumer of DTC genetic testing, we did not need any particular inclusion criteria. However, having the most evolved DTC genetic testing market (Raz et al., 2020), we did limit our DCE to respondents living in the USA. Moreover, including the general population also allows for the investigation of potential consumer groups and differences between them in perceived fairness. The minimum sample size was determined to be 250 with Johnson’s rule of thumb (de Bekker-Grob et al., 2015). Considering a relatively large sample size of 500 or more respondents, the location of respondents, and cost factors, we decided to collect respondent data through a self-administered Internet survey. For the implementation of the Internet survey, we used LimeSurvey Version 4.1.0. We started the survey with a brief introduction of the topic and the task at hand, followed by an exemplary choice set. The choice sets were in a different random order for every respondent to prevent bias (Mangham et al., 2009; McCullough, 2002). Because we applied generic labeling (Service A, Service B, Service C), as opposed to specific labeling (e.g., brand names), shuffling alternatives was not necessary. Moreover, we did not shuffle attribute order due to the partial profile design, which forced respondents to consider different attributes for every choice. Hence, changing their order was not only not required but might have confused participants. To ensure proper completion of the survey and thus ensure data quality, we also added a validation question on the 9th position of the choice sets, resulting in a total of 13 choice sets per respondent. The validation question looked identical to the other choice sets, but instead, the levels of the alternatives were replaced with a text prompting the respondent to skip this question, thus allowing us to filter out all participants that did not look at the attributes at all and randomly chose alternatives. We also added the option to click on any attribute or level to gain information on the attribute and its respective levels. Lastly, we closed the survey by collecting standard (e.g., age, gender) and genetics-specific demographics from the respondents (cf. Table 2). An overview of the survey procedure is provided in Fig. 2. An exemplary choice set as presented during the survey can be found in Supplementary Material 2.

For pretesting our survey, we first conducted a design pretest with fellow researchers, asking for feedback on the survey design, provided information, comprehensibility of the overall choice experiment, and perception of attributes/levels. Overall, 12 researchers provided their feedback, indicating that while the choice experiment with its attributes and levels was comprehensible, the presentation and introduction of the topic needed to be improved. After addressing most design problems, the survey was pretested using the online recruitment system Amazon Mechanical Turk. Of 62 participants, 53 completed the survey, of which 12 failed the validity question, resulting in a conversion rate of 41/62 ≈ 66,1%. Analysis of the pretest data with JMP further confirmed the feasibility of the DCE design.

### Analyzing of discrete choice experiment data

Between the 27^th^ of May 2020 and the 5^th^ of June 2020, 3,077 individuals were asked to participate in our Internet survey via the online recruitment system Access by Cint. Of these, 1,551 (50.41%) dropped out before completion of the survey, and an additional 794 (25.80%) respondents were automatically rejected due to quality termination (i.e., completion of the survey took less than three minutes, or the participant failed the validation question). Lastly, of the successful completes, 55 (7.51%) respondents answered every single question with the non-choice. While this may be a viable preference statement, non-choices do not influence the choice model as they do not provide any additional information on the trade-off of attribute levels (Mangham et al., 2009). Consequently, a total of 677 applicable responses were collected, translating to 8,124 observations (677 respondents × 12 choices) and a conversion rate of 22.00%. To get a representative sample of the US population, invites were managed via Access by Cint to represent the US Census of 2019 for the demographics age and gender. However, factors such as minimum age for survey participation led to minor deviations from the Census. For example, 59.2% of our respondents were female, while the US population had 49.2%, according to the Census. An overview of collected socio-demographic data and their respective distributions, as well as the 2019 Census, are shown in Table 2.

Concluding the data collection, the survey data can be used to estimate an appropriate choice model to analyze consumer fairness perception. Following McCullough (2002) and allowing single utility weights, we defined all attributes, including our numerical attribute *price*, as part-worth attributes. For the choice model, we selected a *conditional logistic regression* to estimate the probability that a configuration is preferred. This is due to the fact that choice modeling requires a linear model based on response attributes, unlike simple logistic regression. Finally, after the model is estimated, various analyses can be performed, such as comparing the relative importance of attributes, ranking different hypothetical alternatives by overall fairness (overall alternative utility) or WTP (Lancsar & Louviere, 2008; Mangham et al., 2009). All estimations and analyses were run with JMP. We present the results of these analyses and discuss them in the following.

**Table 2:** Panel demographics

Demographic	Respondents	N	Ratio%	US Census%
Gender	Male	268	39.6	50.8
	Female	401	59.2	49.2
	Unknown	8	1.2	-
Age	18–29 years	107	15.8	23.2
	30–39 years	126	18.6	17.9
	40–49 years	131	19.4	16.5
	50–59 years	118	17.4	17.1
	60–69 years	118	17.4	15.6
	70–80 years	65	9.6	9.7
	Unknown	12	1.8	-
Ethnicity	American Indian	4	0.6	1.3
	Asian	41	6.1	5.9
	Black or African American	40	5.9	13.4
	Hispanic or Latino	30	4.4	18.3
	Native Hawaiian/ Pacific Islander	1	0.1	0.2
	White/Caucasian (not Hispanic)	534	78.9	60.4
	From multiple races	10	1.5	2.7
	Unknown	17	2.5	-
Known genetic predisposition(s) for disease(s)	Yes - Respondent	33	4.9	-
	Yes - Family Member	27	4.0	-
	Unknown	59	8.7	-
Previously taken genetic test(s)	No Genetic Test taken	571	84.3	-
	Clinical/Medical Test	33	4.9	-
	DTC Lifestyle Test	43	6.4	-
	DTC Health Test	14	2.1	-
	DTC Relationship Test	15	2.2	-
	Unknown	14	2.1	-

## Results

### Utility of attributes

#### Significance of attributes

Analysis of respondent data provides insights into the relative importance and utility of the examined attributes. As can be seen, the model only analyzes the main effects (i.e., interactions are not considered). For an analysis considering two-way interactions including *price*, please refer to Supplementary Material 3. The effect summary (Table 3) shows the attributes in ascending order of their p-values. Transforming the p-value to the LogWorth allows for a more detailed significance analysis. For a better visual representation, the blue line on the bar chart indicates the 0.01 significance level at LogWorth = 2, with larger values indicating higher significance to the model. Since our experiment allowed respondents to reject all business models by choosing the non-choice option, this also needs to be considered by the model. The effect of this choice is shown in Table 3 as *no choice indicator*. Moreover, Table 3 shows that all attributes (effects) except *data storage* (*p* = 0.42) are significant for the choice model at *p* < 0.01 significance level. *Price* is the most significant effect, followed by the *no choice indicator*. Hence, *price* has the largest impact on a respondent’s fairness perception toward a certain business model. In contrast, *data storage* (i.e., how consumers’ genetic data is stored by the provider) has no significant impact on consumer fairness perception.

**Table 3:**
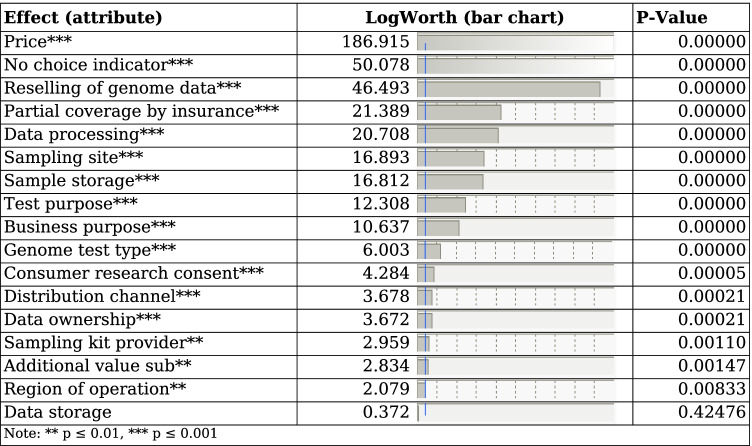
Effect summary of the discrete choice experiment

** *p* ≤ 0.01, *** *p* ≤ 0.001

#### Model comparison criteria

JMP further provides comparison criteria for different models, namely the Akaike’s Information Criterion (AICc), Bayesian Information Criterion (BIC), -2 *Loglikelihood, and − 2*Firth Loglikelihood, with smaller statistics indicating a better fit for the model. The used choice model has an AICc of 20,944.51, a BIC of 21,126.4, a -2 *Loglikelihood of 20,892.33, and a -2*Firth Loglikelihood of 20,683.86. Although the criteria are not comparable amongst each other, a value of the same size indicates a stable model was found. Nonetheless, the values of the criteria are considerably large. The model is potentially still a good fit, as the values tend to increase with additional effects and a larger sample size due to more variation factors (Kass & Raftery, 1995). For example, the DCE mixed logit model reported by Jeong (2017) has an AICc of 7,363 and a BIC of 7,466 with only four attributes and two or three levels each. However, we did not consider different calculation models or models with only a subset of attributes for this research as we are interested in the main effects of all attributes. Moreover, the inclusion of two-way interactions led to similar comparison criteria (see Supplementary Material 3). It should be noted, though, that removing the *no choice indicator* (i.e., the model does not consider when respondents chose the non-option) reduces all criteria values by more than 8,900 (ca. 43%). Because the removal of observations tends to reduce the values of model comparison criteria and our sample consisted of 1949 (23.99%) non-choice observations, this finding is not surprising. For example, as can be seen in Supplementary Material 4, where we use the same choice model for data from only 59 respondents (708 observations), who have taken a genetic test before, the comparison criteria values reduce by about 19,000. Furthermore, since non-choices do not contain any additional information for the model, exclusion may also result in smaller comparison criteria values (Mangham et al., 2009). In contrast to the no choice indicator, removing other effects did not significantly reduce the values of the comparison criteria.

#### Part-worth utilities of attributes

The marginal utility table (Table 4) provides the part-worth utility for every attribute level, indicating its marginal distance from the mean attribute utility. For example, *business purpose - for profit* has a marginal utility value of -0.128. Therefore, the other level from the *business purpose* attribute (*nonprofit*) must have the same distance from the mean (+ 0.128) with a positive impact. Thus, the *nonprofit* level has a positive utility of + 0.256 compared to the *for-profit* level. One notable exception to this is *price*. Since this attribute is numerical and continuous, the estimate describes the negative utility for every unit (i.e., every US Dollar) added. For example, the part-worth utility for the *$100* (*$1000*) level is -0.13 (-1.3) over the *$0* level. However, this also brings the benefit of measuring utility for every price possible, even though it was not modeled in the survey. In addition to the marginal utility, Table 4 also depicts the corresponding marginal probability. It represents the probability that an individual chooses level A of an attribute over level B with all other attributes set to their mean or default level. In the case of *sampling site*, this means, considering this attribute, a respondent will select *home or lab collection* over the other levels with a probability of 39.4%.

**Table 4:** Marginal utility and willingness to pay for level manifestations

Attribute	Level	Marginal Utility		WTP
		Utility	Probability	Price Change
Test purpose	Health test	0.194	0.401	237.61 $
	Lifestyle test	-0.083	0.304	20.86 $
	Relationship test	-0.110	0.296	0.00 $
Business purpose	For-profit	-0.128	0.436	0.00 $
	Nonprofit	0.128	0.564	200.32 $
Region of operation	Local	-0.052	0.474	0.00 $
	Worldwide	0.052	0.526	80.79 $
Consumer research consent	Data not used	0.024	0.340	107.93 $
	Mandatory	-0.114	0.297	0.00 $
	Optional	0.089	0.363	158.70 $
Distribution channel	Healthcare professionals only	0.041	0.346	116.21 $
	Internet only	-0.108	0.298	0.00 $
	Multi-contact service	0.067	0.355	136.69 $
Sampling Site	Home collection	0.051	0.346	221.49 $
	Home or Lab collection	0.182	0.394	324.04 $
	Lab collection	-0.232	0.260	0.00 $
Sampling kit provider	Service provider	0.044	0.347	111.23 $
	Service provider or Third party	0.055	0.351	120.40 $
	Third party	-0.099	0.301	0.00 $
Sample storage	Consumer decision	0.203	0.403	320.31 $
	Mandatory	-0.206	0.268	0.00 $
	Never	0.003	0.330	163.77 $
Genome test type	Genotyping	-0.107	0.298	0.00 $
	Genotyping or Sequencing	0.132	0.378	186.94 $
	Sequencing	-0.025	0.324	64.41 $
Data storage	Database for service provider	-0.033	0.322	0.00 $
	Isolated storage	0.022	0.341	43.32 $
	No storage	0.011	0.337	34.85 $
Data ownership	Consumer	0.070	0.535	108.94 $
	Service provider	-0.070	0.465	0.00 $
Data processing	Basic interpretation	-0.033	0.318	130.07 $
	No interpretation	-0.199	0.269	0.00 $
	Value-added interpretation	0.232	0.414	337.29 $
Additional value subscription	No	-0.061	0.469	0.00 $
	Yes	0.061	0.531	95.90 $
Partial coverage by insurance	No	-0.190	0.406	0.00 $
	Yes	0.190	0.594	298.21 $
Reselling of genome data	No	0.280	0.636	438.23 $
	Yes	-0.280	0.364	0.00 $
Price	For each additional +$1	-0.0013	-	-
No choice indicator	-	-0.473	-	-

The marginal probabilities provide some rather interesting insights for the *test purpose* attribute. Since the marginal probability describes the likelihood of choosing a level over the other levels, we can presume that *health tests* are the most probable tests purchased by consumers with 40%, followed by *lifestyle tests* with 30.4%, and lastly, *relationship tests* with 29.6%. Notably, the highest marginal probability is given for *no reselling of genome data*, with a probability of 63.6% for consumers choosing this level over the other.

#### Willingness to pay analysis

When the *price* attribute (or any numerical attribute) is defined as continuous, this allows for calculating the WTP. Since our survey did not ask for a preference to purchase but the preference for fairness, the WTP analysis can be seen as the monetary trade-off for a fairer attribute level. Utilizing the unfairest business model as the baseline, the WTP analysis can unveil how much a consumer is willing to pay for a fairer level of each attribute.

For example, according to our analysis, a potential customer values the *business purpose* of the service provider with $200.32 if it is *nonprofit* instead of *for-profit*. Consequently, the fairest business model has a value of $3,087.69 to consumers (with the unfairest set to a WTP of $0.00). Table 4 shows the WTP price change for every attribute level. WTP analysis provides some interesting insights into consumer value and pricing. First, consumers rate an additional value subscription equally to $95.90. When considering a typical subscription, such as ancestryDNA’s US Discovery ($24.99), this accounts for roughly four months of access to their ancestry network. Second, attributes concerning security and sharing of the consumers’ genetic data are valued highly if they preserve the customers’ privacy, such as not reselling the genetic data ($438.23), not using data for research ($107.93 or $158.70 for freedom of decision), or not storing the DNA sample ($163.77 or $320.31 for freedom of decision). Last, consumers value genome sequencing at $64.41 over the baseline genotyping.

### Utility of direct-to-consumer genetic testing business models

Given the part-worth utilities, the overall utility of a (hypothetical) business model is simply calculated by adding the part-worth utilities of every selected level together. This allows for a comparison of business models in terms of overall perceived fairness by consumers. Consequently, the fairest business model with the attribute levels shown in Table 5 has the maximum utility of 1.956. In contrast, the unfairest business model has a utility of -3.266. The respective attribute levels are also shown in Table 5. It should be noted that including the *no choice indicator* (i.e., respondents are allowed to refrain from selecting any business model) causes a constant additional negative utility of -0.4730 to the overall utility (cf. Table 4).

**Table 5:** Fairest and unfairest hypothetical business model

Attribute	Fairest	Unfairest
Utility	1.95601	-3.26602
Price	$0	$1000
Reselling of genome data	No	Yes
Partial coverage by insurance	Yes	No
Data processing	Value-added interpretation	No interpretation
Sampling site	Home or Lab collection	Lab collection
Sample storage	Consumer decision	Mandatory
Test purpose	Health test	Relationship test
Business purpose	Nonprofit	For-profit
Genome test type	Genotyping or Sequencing	Genotyping
Consumer research consent	Optional	Mandatory
Distribution channel	Multi-contact service	Internet only
Data ownership	Consumer	Service provider
Sampling kit provider	Service provider or Third party	Third party
Additional value sub	Yes	No
Region of operation	Worldwide	Local
Data storage	Isolated storage	Database for service provider

Naturally, the fairest business model represents all the attribute levels that benefit consumers financially or service-wise the most, such as *price* $0, no *reselling of genome data*, a health *test purpose*, and a nonprofit *business purpose*. On the contrary, the unfairest business model costs $1000, does resell genetic data, offers a relationship test, and has a for-profit *business purpose*.

To further examine the utility of DTC genetic testing business models, we have conducted a brief investigation of real DTC genetic testing service providers and the utility they would have according to our choice model. For this, we have investigated two service providers per archetype found by Thiebes et al. (2020) in Supplementary Material 5.

## Discussion

### Principal findings

Analysis of the DCE results delivers interesting insights into consumer perceptions of DTC genetic testing and the fairness of their business models. While our DCE provides hypothetical fairest and unfairest business models, these are highly unlikely to exist in a real-world setting (e.g., a nonprofit company offering a genotyping or sequencing health test through a worldwide multi-contact service for $0). This finding, however, is not surprising, as consumers naturally prefer the best service for the least amount of cost. Thus, it is necessary that consumers and service providers make certain trade-offs to find a balance between the service offered and the costs involved. Nonetheless, overall, the hypothetical models strengthen the notion of consumers’ preference for more privacy-preserving services. In a similar vein, the results from our analysis of real DTC genetic testing business models suggest that privacy-preserving services found in archetype two (high-privacy DTC genomics for enthusiasts) in general achieve a higher utility than the similar test from archetype one (low-cost DTC genomics for enthusiasts). However, as the name suggests, this trade-off comes with a price difference, as the service providers from archetype two are more expensive. Another interesting finding highlighting how price is the most important factor when considering the fairness of DTC genetic testing business models is that consumers seem to prefer simple health tests (archetype four) over comprehensive tests and low data processing (archetype six) in terms of utility, mainly because these health tests are more expensive and often require additional interpretation.

Closer inspection of the part-worth utilities highlights certain business practices consumers seem to perceive to be fair or unfair, respectively. Our results show that marginal utility for genetic data privacy-related attributes is relatively high. In particular, consumers prefer when their genetic data is not resold to third parties, research participation is either optional or not existing, and sample storage is either optional or not existing. *Reselling of genome data* is the attribute with the third-highest significance (LogWorth = 46.493). This is in line with previous research, which shows that consumers desire transparent and privacy-preserving business models (e.g., Lewis et al., 2013; Phillips, 2017) and perceive business models reselling their genetic data as unfair (Raz et al., 2020). This finding is not unexpected as previous research also shows consumers’ concerns about sharing and privacy of their genetic data (e.g., Critchley et al., 2017; Lewis et al., 2013). Further, these results are in line with a recent DCE study on consumer preference for DTC genetic testing policies in Korea, showing similar utility results for privacy-preserving tests (Jeong, 2017). The WTP analysis further strengthens these observations, unveiling that consumers, on average, value not selling their genetic data for revenue equal to $438.23. Adding to this, Briscoe et al. (2020) found that if consumers are to share their data from a typical DTC genetic test, they are expecting to receive a median net payment of $95 (already having deducted the usual costs of the DTC genetic test). Therefore, we can assume that consumers from this study would be willing to pay $95 plus the typical price for a DTC genetic test (ca. $100-$150) if their data is not shared. Although this price is still substantially below the WTP of our results, it further strengthens the notion that consumers perceive genetic testing as fairer if their genetic information is not available to third parties. Adding to this, Tsai et al. (2011) found that consumers are sometimes willing to pay a higher price for goods purchased from privacy-protective online vendors once made aware of their respective privacy policies. Since in the DTC genetic testing market, consumers are also often unaware of service providers’ privacy policies, this may also explain why our findings show a more significant monetary value for not selling genetic data for revenue, as our study made respondents aware of this business practice.

The proposition that consumers perceive privacy-preserving DTC genetic testing business models as fairer is opposed by the fact that *price* (LogWorth = 186.915) is the most significant factor for consumers’ perception of fairness. Therefore, it is likely that consumers prefer lower costs over higher overall service quality. The part-worth utility from $0 to $1000 (-1.3) vastly outperforms every other attribute. Moreover, our additional analysis of two-way interactions including price (cf. Supplementary Material 3) showed that there only exists one significant interaction between *genome test type* and *price*, with most other interactions having little to no significance. This, however, is not surprising, as the test type (e.g., genotyping vs. whole genome sequencing) is mainly responsible for the costs that occur for a service provider while also determining test comprehensibility. Hence, it seems plausible that consumers take the interaction of genome test type and price into account when determining fairness perception. The third most significant attribute is *reselling of genome data*, which only contributes − 0.56 negative utility impact if genetic data is resold. This is in line with findings on willingness to disclose personal genetic data, which show that consumers are willing to provide access to their personal (genetic) health information for monetary incentives (Anderson & Agarwal, 2009; Jeong, 2017) also found that consumers are most sensitive to the pricing of DTC genetic testing, confirming our findings. Further, studies found consumers to be more willing to share personal information when informed about a vendor’s privacy practices and perceiving the business as a whole as fair to them (Dinev & Hart, 2006). These could be just two explanations (amongst many others) for why service providers that should be perceived to employ unfair business practices remain popular to this day. At the same time, the *no choice indicator* (LogWorth = 50.078) is the second most significant effect of the model. It could therefore be assumed that many consumers opt to refrain from DTC genetic testing altogether, as they do not perceive it to be a fair and valuable business market.

To investigate whether consumers who have taken a DTC genetic test before exhibit different fairness perceptions, we further conducted a separate analysis of 59 respondents who stated having taken a DTC genetic test (see Supplementary Material 4 for detailed analysis). In contrast to the main analysis, here *reselling of genome data* (LogWorth = 11.855) has a higher significance than the *no choice indicator* (LogWorth = 7.499). Because these respondents have taken or purchased a DTC genetic test before, it makes sense that they may be more open to perceiving DTC genetic testing business models as fair or fair enough to purchase, thus explaining the lower significance of the *no choice indicator*. On the other hand, having provided their genetic data previously, these respondents also may be more concerned about the reselling of their own genetic data, explaining the higher significance of *reselling of genome data*. Together with the large number of non-choices (ca. 23.99%) for the entire US population-centered respondent sample, these results suggest that certain consumer groups, certain age or ethnicity groups, might show a high aversion toward DTC genetic testing in general, while others are more adept.

Our DCE also resulted in some surprising findings regarding selected attributes. For *sampling site*, the *lab collection* is disliked by consumers. While *lab collections* allow for the involvement of healthcare professionals and should thus indicate a higher level of quality, consumers might deter from *lab collections* due to inconvenience or rejection of medical professional involvement. As previous research has stated, DTC genetic testing originated from self-responsible health and a do-it-yourself mindset (Allyse et al., 2018). *Home collections* are the purest form of this, as the consumer neither comes into contact with a healthcare professional nor lab staff. Additionally, because our study was conducted at the beginning of the COVID-19 pandemic in 2020, recommendations to minimize social interactions may have also been a driver for respondents to disavow *lab collections* at the time. The marginal utility table clearly shows that consumers are interested in DTC health tests. Briscoe et al. (2020) also find personal health to be an important driver for DTC genetic testing. These findings may indicate that while DTC genetic testing has advanced into the DTC 2.0 era (Allyse et al., 2018), consumers are yet to adapt to personalized health. Moreover, we found insignificance of *data storage* (LogWorth = 0.372). This may indicate that, while consumers do not want their data to be sold, they are not bothered if the service provider stores and uses their genetic data for service improvements. Although internal data usage does not involve the sharing of data with third parties, these practices can as well pose a threat to personal genetic privacy. Therefore, these results somewhat deviate from previous research on consumers’ concerns regarding their privacy and indicate that not all genetic privacy attributes are important for the fairness perception of DTC genetic testing. Finally, the significance of *region of operation* (LogWorth = 2.079) suggests that consumers do not mind whether the service provider operates only locally or worldwide (i.e., potentially from another country). This stands in conflict with findings of previous studies (e.g., Lewis et al., 2013; Majumder et al., 2016), signifying that purchasing services not residing in the own country may be a liability due to different regulations and less strict governance (Lewis et al., 2013). However, since most DTC genetic testing service providers operate from the USA (Phillips, 2018), the low significance may be caused by the regional limitation of our survey, as in most cases, the DNA sample will not leave the USA even when the provider operates worldwide.

### Contributions to research and practice

This study conveys several contributions to research (Table 6) and practice (Table 7). For research, we contribute to the literature streams on consumer perceptions of DTC genetic testing and fairness by contextualizing the retail fairness concept to the DTC genetic testing space. Our findings showcase a better understanding of which services consumers favor and what attributes influence preference. We show that *price* is the main driver for consumers’ perceived fairness of DTC genetic testing services’ business models. While we recognize that prior research has focused on the fairness of genetic testing in general (e.g., de Vries et al., 2015; Morrow, 2009) and to a lesser extent in DTC genetic testing (e.g., Phillips, 2017), these studies do not investigate consumers fairness perception of DTC genetic testing business models, but rather focus on ethical or regulatory concerns. To this end, we provide a first look into fairness perceptions of DTC genetic testing business models. In doing so, our research adds to our understanding of retail fairness in this market segment, as well as to our understanding of the importance of fairness and justice perceptions in disruptive, digital (healthcare) business models. Further, this study offers a choice model for DTC genetic testing business models, unveiling 16 relevant attributes with a total of 42 levels and their respective utilities for perceived fairness. This choice model permits novel insights into the DTC genetic testing landscape, allowing us to explain and predict consumers’ perceived fairness of DTC genetic testing business models. Highlighting that consumers, broadly speaking, seem to perceive privacy-preserving business practices fairer than non-privacy-preserving business practices, this study also adds to the literature on organizational privacy assurances and consumer behaviors. Especially, it extends our understanding of the impact of privacy-preserving business practices on consumers’ perceived fairness of DTC genetic testing business models by providing a first overview of DTC genetic testing business model attributes and levels relevant to service fairness. We also provide minor adjustments (i.e., splitting *fee type* into *price* and *additional value subscription* or changing combination attributes to consumer choice attributes, such as *sampling site* and *test purpose*) to extend the taxonomy of Thiebes et al. (2020) to a viewpoint on the fairness of business models. These alterations could also prove valuable to investigating DTC genetics on a per-test rather than a service provider basis.

**Table 6:** Summary of key contributions to research

Previous gap in research	Key contributions
Lack of understanding of business practices in the DTC genetic testing space impact consumers’ perceived fairness of DTC genetic testing business models.	Contextualization of the retail fairness concept to DTC genetic testing, adding to our understanding of retail fairness in this market segment, as well as to our understanding of the importance of fairness and justice perceptions in digital (healthcare) business models in general.
	Choice model explaining and predicting consumers’ perceived fairness of DTC genetic testing business models. Adding to the literature on organizational privacy assurances and consumer behaviors, in particular, extending our understanding of the impact of privacy-preserving business practices on consumers’ perceived fairness of DTC genetic testing business models.
DCEs are primarily used to elicit response variables related to purchasing intentions (e.g., WTP, perceived attribute importance).	Demonstration of DCEs as an appropriate method to elicit consumers’ fairness perceptions, especially regarding digital business models (in healthcare). Showing that consumers’ perceived fairness can be captured through choice preference.

**Table 7:** Summary of key contributions to practice

Stakeholders	Key contributions
Consumers	• With our developed choice model, consumers may assess the perceived fairness of DTC genetic testing service providers before purchase. • Our results inform consumers about important aspects of DTC genetic testing business models, such as reselling genetic data and which trade-offs other consumers might be willing to take for a lower price.
Service providers	• With our developed choice model, service providers may analyze and adapt their business models in terms of consumer fairness. • The DCE outlines which business model attributes are of high importance to consumers’ perceived fairness.
Policymakers	• Understanding of perceived fairness for DTC genetic testing business models allows policymakers to create fair and informed regulations, protecting consumers while ensuring a free market economy. • The choice model can help uncover consumers’ genuine desires for DTC genetic testing and allows addressing of ethical and legal concerns

From a practical perspective, our research yields important implications for consumers, service providers, and policymakers alike. For consumers, our results may serve as a source of information about the relevant characteristics of DTC genetic testing models that determine fairness perceptions. This can be particularly relevant when consumers strive to make an informed decision for or against sharing their personal genetic data with such services. For DTC genetic testing service providers, the choice model presented in this paper provides insights into which aspects of business models are relevant to consumers and how they influence their fairness perceptions. The model can serve as a valuable tool for service providers when assessing consumers’ fairness perceptions of their own business model and comparing it with the business models of competitors. Our findings also allow service providers to adapt their own business models to consumers’ preferences and strengthen their position in the DTC genetic testing market. For example, it may be a viable option for DTC genetic service providers to adopt a more privacy-preserving business model and make consumers aware of their privacy policies, even if this means paying a premium for consumers, to increase perceived fairness and customer satisfaction. Moreover, our results could help policymakers create fair and informed regulations for the DTC genetic testing market, protecting consumers while ensuring a free market economy. Potential ethical and legal concerns (e.g., Hendricks-Sturrup & Lu, 2019; Majumder et al., 2016; Raz et al., 2020) can be addressed by understanding what consumers genuinely desire of DTC genetic testing and what impact business model decisions have on customer satisfaction. We further provide a hypothetical fairest and unfairest business model, which, while being highly unrealistic, can be used to manage trade-offs between services offered, pricing, and consumer dissatisfaction influence.

### Limitations and future research

The limitations of this study are as follows. First, the selection of attributes and levels is largely reliant on previous work by Thiebes et al. (2020). Thus, it is possible that important attributes for the perceived fairness of DTC genetic testing business models were not considered, while other less relevant attributes are included. For example, the DCE on consumer preference of DTC genetic testing policies by Jeong (2017) includes an attribute concerning the number of genetic markers observed per test. Future research should therefore investigate the model in terms of relevant attributes and levels. Second, respondent data might not be representative of the consumer landscape of DTC genetic testing. While aimed to obtain a sample resembling the US Census, socio-demographics show this is not the case for all data. Also, the USA-only regional restriction of participants, together with our low response rate of 22%, might have caused some skewed results. Additionally, our sample size of 637 might be limiting for our study design with 144 choice sets. Consequently, attempting replication of our approach, perhaps with respondents from other regions or a magnitude of respondents, could capture a more representative sample of consumers. Third, we primarily considered the main effects for this study, as the high number of levels already allows for 256 effects when considering two-way interactions and main effects. This, however, would require identifying meaningful interactions to retain the comprehensibility of the model. While we conducted a brief investigation of two-way interactions including price (cf. Supplementary Material 3), the majority of possible interactions were not considered. Hence, it is possible that important interactions between attributes were not discovered. Last, the quality and design of the model might exhibit some limitations. Compared to typical DCEs, we have a rather large number of attributes and choice sets. The use of a partial profile design with blocking might have overwhelmed participants and thus influenced data quality. Future research should also compare the resulting model to different alternative models, such as hierarchical Bayes, which could provide new or improved insights into the perceived fairness of DTC genetic testing business models.

Building on our work, future research should further investigate the fairness of DTC genetic testing business models. One possible approach for this would be a closer investigation of the overall utility of prevailing business models or business model archetypes of DTC genetic testing. Because our study suggests that many consumers do not yet use DTC genetic testing services and additional analysis of respondents who have taken a DTC genetic test before outlines possible differences in consumer groups (see Supplementary Material 5), a consumer segmentation by means of the DCE could be used as a first starting point to identify how and if fairness perception is influenced by demographics such as age, ethnicity, income or prior usage of genetic testing services.

## Conclusions

The DTC genetic testing market is a fast-growing business sector and is likely to gain more importance over time. With an increasing demand for personal healthcare and curiosity about oneself, DTC genetics has the potential to lastingly impact human genetic testing. However, with DTC genetic testing being relatively young, many concerns and problems are yet to be answered. This paper provides a first overview of consumers’ perceived fairness of DTC genetic testing business models by conducting a DCE to elicit preference. In doing so, we construct a choice model consisting of 16 relevant attributes and 42 levels of business models in genetic testing. Therefore, our research provides a tool for evaluating and comparing the influence of hypothetical business models on the perceived fairness of consumers. Moreover, this study investigated retail fairness in DTC genetic testing, providing novel insights on consumers’ views for research, service providers, and regulatory authorities.

## Supplementary Information

Below is the link to the electronic supplementary material.

ESM 1 (PDF 113 KB)

ESM 2 (PDF 126 KB)

ESM 3 (PDF 160 KB)

ESM 4 (PDF 150 KB)

ESM 5 (PDF 214 KB)
