# Supplementary material for: Perceived fairness of direct-to-consumer genetic testing business models
Source: Electron Mark. 2022 Jul 18;32(3):1621–38. doi: 10.1007/s12525-022-00571-x (PMC9294841; doi:10.1007/s12525-022-00571-x)
Supplement: Supplementary file 1 — (PDF 113 KB) [file 12525_2022_571_MOESM1_ESM.pdf]

Electronic Markets
The International Journal on Networked Business

Perceived Fairness of Direct-to-Consumer Genetic Testing Business Models

Supplementary Material 1

List of Utilized Attributes and Levels

Table S1-1 provides an overview of all attributes and levels utilized for the present study. It further entails a rationale for each attribute and a more detailed description of each level. During the survey, respondents were offered these rationales and level descriptions when clicking on an attribute for further information.

Dimension	Rationale	Level
Test purpose	What is the purpose of the genetic test?	**Health tests:** Genetic tests aiding persons with medical conditions or risk groups with identifying and/or preventing health issues (e.g., diabetes treatment, drug tolerability, or cancer prevention).
		**Lifestyle tests:** Genetic tests mainly focused on individuals who are enthusiasts and seek insight into their genetics. Common test types include ancestry, genetic dating, trait testing, or wellness and sports analysis.
		**Relationship tests:** Genetic tests that require the DNA of two people and determine whether and how these two individuals are related (e.g., immigration due to relations, maternity test, or paternity test).
Business purpose	Does the service provider intend to make a profit from the service?	**For-profit:** The business currently makes or intends to make a profit from the service offered, either directly from the price the consumer pays or through indirect income (e.g., reselling genome data).
		**Non-profit:** The business does not make a profit from the service and only generates income to maintain and continue offering the service to the public. Also, it usually contributes to genetic research.
Region of operation	Where is the service available?	**Local:** The service is only available in the country the service provider is registered in.
		**Worldwide:** The service is offered all over the world except for countries that do not allow such tests by law.
Consumer research consent	Is the consumer's genetic data used for research?	**Data not used**: The consumer's genetic data is not used for research purposes.
		**Mandatory:** By choosing the service, the consumer automatically agrees that the consumer's genetic data is used for research.
		**Optional:** The consumer decides whether genetic data is used for research purposes or not. This decision can be revoked at any time.
Distribution channel	How is the service distributed to the consumer?	**Healthcare professionals only:** A healthcare professional is required and serves as a means for distributing and, in some cases, carrying out sample collection and follow-up patient counseling but is neither responsible for performing the actual genetic testing nor for interpreting test results.
		**Internet only:** All service provider to consumer communication is done via the internet (i.e., the service provider's website).

Editorial Office:
c/o Information Systems Institute
Leipzig University
www.electronicmarkets.org

Grimmaische Str. 12
04109 Leipzig, Germany
editors@electronicmarkets.org

Dimension	Rationale	Level
		**Multi-contact service:** This includes internet solutions, mobile apps, telephone consulting, stores, and home visits to offer the service to the consumers.
Sampling Site	Where is the DNA sample collected?	**Home collection:** The collection kit is mailed to the consumer's home. The sample is then taken by the consumer themselves (e.g., buccal swab or saliva sample) and sent back for analysis.
		**Lab collection:** The consumer needs to visit a lab where the sample is taken by the staff. Lab collection is usually offered either as a convenience to the consumer or because it is legally required (e.g., a paternity test that is to be acknowledged by the court).
		**Home or Lab collection:** The provider offers both options for the service, and the consumer may choose the sampling site.
Sampling kit provider	Who provides the sampling kit?	**Service provider:** The service provider offers the sampling kit.
		**Third party:** The sampling kit is provided by a third party (e.g., another service provider).
		**Service provider or Third party:** The service provider offers both options for the offered service, and the consumer may choose the sampling kit provider.
Sample storage	Is the DNA sample stored?	**Mandatory:** The sample is stored either because the service provider decides to do so or because it is legally required (e.g., legally binding paternity test).
		**Never:** The sample is destroyed after analysis.
		**Consumer decision:** The consumer may decide whether and for how long the sample is stored. This might come with additional costs.
Genome test type	What method is used to analyze the sample?	**Genotyping:** Only very few selected regions of the genome are sequenced (e.g., SNPs) and then compared to another individual's genome or database of multiple genomes. When the region of interest is known, this allows for minimal sequencing effort to gain the information desired.
		**Sequencing:** The whole genome/exome is sequenced, i.e., the whole sequenced genome is available for analysis. Of course, a comparison of regions of interest is still possible, but the majority of the sequenced DNA (high effort and high data capacity) is usually not touched but available, e.g., for future testing.
		**Genotyping or Sequencing:** The consumer may decide whether the DNA sample is genotyped or sequenced.
Data storage	How is the genetic data stored after analysis?	**Database for service provider:** The produced genetic data is stored in a common database, which is used to improve service quality (e.g., more data for genotyping comparison of future consumers).
		**Isolated storage:** The genetic data is stored by the service provider but only available to the consumer. The consumer also decides for how long it is stored. This might come with additional costs.
		**No storage:** The genetic data is deleted shortly after the consumer retrieves the genetic data.
Data ownership	Who owns the produced genetic data?	**Consumer:** The consumer keeps all rights to their genetic data.
		**Service provider:** The service provider claims the rights to the genetic data.
Data processing	How is the genetic data interpreted?	**No interpretation:** The produced genetic data is not interpreted. Only the generated raw genetic data is offered to the consumer.
		**Basic interpretation:** The genetic data is interpreted in a basic manner. The consumer usually receives a report on certain information (e.g., ancestry information, health traits, paternity test, or cancer test).

Dimension	Rationale	Level
		**Value added interpretation:** The interpretation is augmented with additional services such as, e.g., carrying out legally binding paternity tests (as opposed to cheaper, not legally binding tests) or providing diet plans and/or supplements based on the analysis of the consumer's genome.
Price	How much does the service cost?	**$0:** There are no (monetary) costs for the service.
		**$100:** The service costs $100.
		**$1000:** The service costs $1000.
Additional value subscription	Are additional services available through a subscription?	**Yes:** Additional services such as sample storage, data storage, data access, community access, diet plan, or supplement subscription are offered with a post-test subscription.
		**No:** No additional services are offered with a post-test subscription.
Partial coverage by insurance	Can the costs for the service be partially covered by health insurance?	**Yes:** Health insurance can partially cover the consumer's costs for the service.
		**No:** Health insurance will not cover the consumer's costs for the service.
Reselling of genome data	Does the service provider sell the consumer's genetic data?	**Yes:** The consumer's genetic data is sold to a third party.
		**No:** The consumer's genetic data is not sold to a third party.

Table S1-1: Attributes, rationale, and level descriptions of DTC genetic testing business models
