# Supplementary material for: Perceived fairness of direct-to-consumer genetic testing business models
Source: Electron Mark. 2022 Jul 18;32(3):1621–38. doi: 10.1007/s12525-022-00571-x (PMC9294841; doi:10.1007/s12525-022-00571-x)
Supplement: Supplementary file 2 — (PDF 126 KB) [file 12525_2022_571_MOESM2_ESM.pdf]

Electronic Markets
The International Journal on Networked Business

Perceived Fairness of Direct-to-Consumer Genetic Testing Business Models

Supplementary Material 2

Exemplary Choice Set

Figure S2-1 depicts an exemplary choice set from the twelfth block (choice set 140/144). Respondents are given a short task description followed by a table showing three business models from the generic Service A, Service B, and Service C. Six of the sixteen attributes have varying levels, namely *business purpose, region of operation, sampling kit provider, genome test type, data storage,* and *additional value subscription*. The remaining ten share the same level between the three choices. As shown for the *business purpose* attribute clicking on an attribute will open a more detailed description of the attribute and its possible levels (see Supplementary Material 1 for all descriptions). Respondents may choose Service A, Service B, or Service C to have the fairest business model. Alternatively, they may deem all business models unfair by clicking *None*. In this example, the respondent chose Service A.

Editorial Office:
c/o Information Systems Institute
Leipzig University
www.electronicmarkets.org

Grimmaische Str. 12
04109 Leipzig, Germany
editors@electronicmarkets.org

Your task:

You see **3** business models below. Please select the business model that **you** think is the fairest. If you think none of the presented business models are fair, you may also choose the ***None*** option instead.

Which business model do you consider to be the fairest?

Click on attribute to open help	Service A	Service B	Service C
▸ Test purpose	Relationship test		
▾ Business purpose	Non-profit	For profit	Non-profit
**Does the service provider intend to make profit from the service?**<br>**For-profit:** The business currently makes or intends to make a profit from the service offered, either directly from the price the consumer pays or through indirect income (eg. reselling genome data).<br>**Non-profit:** The business does not make profit from the service and only generates income to maintain and continue offering the service to the public. Also, it usually contributes to genomics research.			
▸ Region of operation	Local	Worldwide	Local
▸ Consumer research consent	Optional		
▸ Distribution channel	Internet only		
▸ Sampling site	Home collection		
▸ Sampling kit provider	Service provider or Third party	Third party	Service provider
▸ Sample storage	Consumer decision		
▸ Genome test type	Genotyping or Sequencing	Sequencing	Genotyping
▸ Data storage	No storage	Isolated storage	Database for service provider
▸ Data ownership	Service provider		
▸ Data processing	Value added interpretation		
▸ Price	$1000		
▸ Additional value subscription	No	Yes	No
▸ Partial coverage by insurance	Yes		
▸ Reselling of genome data	Yes		

Service A | Service B | Service C | None

Figure S2-1: Exemplary choice set
