# Supplementary material for: Perceived fairness of direct-to-consumer genetic testing business models
Source: Electron Mark. 2022 Jul 18;32(3):1621–38. doi: 10.1007/s12525-022-00571-x (PMC9294841; doi:10.1007/s12525-022-00571-x)
Supplement: Supplementary file 3 — (PDF 160 KB) [file 12525_2022_571_MOESM3_ESM.pdf]

Electronic Markets
The International Journal on Networked Business

Perceived Fairness of Direct-to-Consumer Genetic Testing Business Models

Supplementary Material 3

Analysis of Two-way Interactions Including Price

Having determined that *price* was the most significant attribute of our choice model, considering only main effects, we also conducted analysis of a choice model considering main effects and all possible two-way interactions with *price* (i.e., all other 15 attributes interacting with the former). Apart from the addition of the two-way interactions, the analysis was performed identical to the main choice model. The choice model analysis has an AICc of 20,946.54, a BIC of 21,296.04, a -2*Loglikelihood of 20,845.91, and a -2*Firth Loglikelihood of 20,163.28.

Significance of Attributes

As can be seen from the effect summary shown in Table S3-1 the model significances are similar to the main choice model, which is to be expected as both models rely on the same data and attributes. As such, *price* is still the most significant attribute and has the largest impact on respondent's fairness perception. Moreover, the *no choice indicator* is still the second most significant effect followed by *reselling of genome data* at third position. Two noteworthy deviations to the effect order by significance are *partial coverage by insurance* and *consumer research consent*, which both score 3 and 5 positions lower than in the main choice model. However, as the LogWorth values for neighboring effect are very close in both models such minor changes are to be expected. Further, due to the addition of 15 new effects (i.e., the two-way interactions), the attributes *distribution channel*, *consumer research consent*, and *sampling kit provider* no longer bare significance.

Regarding the added two-way interactions, most of these interactions are not significant, having lower LogWorth values than any main effect. Therefore, this model suggests, that there are no significant interactions for these attributes and *price*. The only exception to this is the interaction of *genome test type* and *price*, which is significant at the $p < 0.01$ level. One possible explanation for this is that the test type (i.e., genotyping, sequencing, or both) is a main factor for determining the costs that occur for the service provider, while also

impacting how comprehensive the test results may be for the customer. It is thus plausible, that this dependence of product and costs is mirrored in the significant interaction between both attributes.

Effect (attribute)	LogWorth (bar chart)	P-Value
Price***	142.964	0.00000
No Choice Indicator***	45.188	0.00000
Reselling of genome data***	37.001	0.00000
Data processing***	15.899	0.00000
Sampling Site***	13.019	0.00000
Sample storage***	11.57	0.00000
Partial coverage by insurance***	11.042	0.00000
Test purpose***	10.296	0.00000
Business purpose***	6.744	0.00000
Genome test type***	6.16	0.00000
Data ownership***	4.238	0.00006
Additional value sub**	2.881	0.00132
Genome test type×Price**	2.637	0.00231
Region of operation**	2.481	0.00330
Partial coverage by insurance×Price	1.974	0.01061
Distribution channel	1.842	0.01438
Additional value sub×Price	1.681	0.02084
Consumer research consent	1.553	0.02796
Sampling kit provider	1.221	0.06005
Sampling Site×Price	0.929	0.11764
Region of operation×Price	0.82	0.15145
Reselling of genome data×Price	0.694	0.20218
Data storage	0.57	0.26894
Business purpose×Price	0.551	0.28132
Data processing×Price	0.543	0.28657
Data ownership×Price	0.507	0.31129
Consumer research consent×Price	0.468	0.34004
Sampling kit provider×Price	0.455	0.35071
Data storage×Price	0.447	0.35750
Distribution channel×Price	0.304	0.49661
Test purpose×Price	0.172	0.67315
Sample storage×Price	0.106	0.78281
Note: ** $p \leq 0.01$, *** $p \leq 0.001$		

Table S3-1: Effect summary of choice model with two-way interactions including price

Part-Worth Utilities of Attributes

The marginal utility table (Table S3-2) provides the part-worth utility for every attribute level, indicating its marginal distance from the mean attribute utility. Additionally, Table S3-2 also entails the marginal probability (i.e., the probability that an individual chooses level A of an attribute over level B with all other attributes set to their mean or default level). As this choice model also includes all attributes as main effects and analysis was performed on the same dataset, both marginal utility and marginal probability do not deviate substantially from

the main choice model. This once again shows that both models are similar in how attributes and their respective levels contribute to respondents' fairness perception.

**Attribute**	**Level**	**Marginal Utility**	
		**Utility**	**Probability**
Test purpose	Health test	0.200	0.403
	Lifestyle test	-0.096	0.300
	Relationship test	-0.104	0.297
Business purpose	For profit	-0.137	0.432
	Nonprofit	0.137	0.524
Region of operation	Local	-0.048	0.476
	Worldwide	0.048	0.524
Consumer research consent	Data not used	0.022	0.336
	Mandatory	-0.107	0.299
	Optional	0.086	0.362
Distribution channel	Healthcare professionals only	0.036	0.344
	Internet only	-0.114	0.297
	Multi-contact service	0.078	0.359
Sampling Site	Home collection	0.027	0.338
	Home or Lab collection	0.190	0.397
	Lab collection	-0.216	0.265
Sampling kit provider	Service provider	0.057	0.352
	Service provider or Third party	0.042	0.347
	Third party	-0.100	0.301
Sample storage	Consumer decision	0.213	0.407
	Mandatory	-0.208	0.267
	Never	-0.006	0.327
Genome test type	Genotyping	-0.113	0.296
	Genotyping or Sequencing	0.119	0.374
	Sequencing	-0.006	0.330
Data storage	Database for service provider	-0.030	0.324
	Isolated storage	0.011	0.337
	No storage	0.019	0.340
Data ownership	Consumer	0.075	0.538
	Service provider	-0.075	0.462
Data processing	Basic interpretation	-0.019	0.322
	No interpretation	-0.200	0.269
	Value-added interpretation	0.219	0.409
Additional value subscription	No	-0.039	0.481
	Yes	0.039	0.519
Partial coverage by insurance	No	-0.204	0.399
	Yes	0.204	0.601
Reselling of genome data	No	0.280	0.637
	Yes	-0.280	0.367
Price	For each additional +$1	-0.0014	-
No choice indicator	-	-0.458	-

Table S3-2: Marginal utility and probability of choice model with two-way interactions including price
