# Supplementary material for: Perceived fairness of direct-to-consumer genetic testing business models
Source: Electron Mark. 2022 Jul 18;32(3):1621–38. doi: 10.1007/s12525-022-00571-x (PMC9294841; doi:10.1007/s12525-022-00571-x)
Supplement: Supplementary file 4 — (PDF 150 KB) [file 12525_2022_571_MOESM4_ESM.pdf]

Electronic Markets
The International Journal on Networked Business

Perceived Fairness of Direct-to-Consumer Genetic Testing Business Models

Supplementary Material 4

Analysis of Respondents who Have Taken a Direct-to-Consumer Genetic Test

To investigate whether having taken a DTC genetic test influences fairness perception of DTC genetic testing business models, we also conducted an analysis only including respondents who stated that they had taken at least one DTC genetic test before. This included either a DTC lifestyle test (43 respondents), DTC health test (14 respondents), or DTC relationship test (15 respondents), but not clinical/medical test. Because some respondents declared having taken multiple tests, our final dataset consists of 59 respondents, having taken at least one DTC genetic test before. Analysis was performed analogue to the main analysis using the identical choice model, which only considers main effects. The choice model analysis has an AICc of 1,786.21, a BIC of 1,902.77, a -2*Loglikelihood of 1,732.15, and a -2*Firth Loglikelihood of 1,588.40.

Significance of Attributes

The effect summary (Table S4-1) provides interesting insights into the fairness perception of respondents who have taken a DTC genetic test before. First, only five effects are significant namely, *price*, *reselling of genome data*, *no choice indicator*, *data processing*, and *sampling site*, which are all significant at the p < 0.001 significance level. However, the insignificance of many remaining effects is most likely to the rather small sample size of 59 respondents as Johnson's rule of thumb (de Bekker-Grob, Donkers, Jonker, & Stolk, 2015) suggests a minimum sample size of 250 for our DCE. *Price* is once again the most significant attribute, suggesting that it is also the main driver for fairness perception of respondents who have taken a DTC genetic test before. Deviating from the main analysis of all respondents *reselling of genome data* (LogWorth=11.855) is more significant to the fairness perception than the no choice indicator (LogWorth=7.499). Because these respondents have taken or purchased a DTC genetic test before, it makes sense that they may be more open to perceiving DTC genetic testing business models as fair or fair enough to purchase, thus explaining the lower

significance of the no choice indicator, which indicates that respondents refrained from perceiving any of the presented business models as fair. On the other hand, having provided their genetic data previously, these respondents also may be more concerned about reselling of their own genetic data, explaining the higher significance of *reselling of genome data*. Another interesting change is that partial coverage by insurance is only the 8th most significant effect compared to it the main analysis where it is 4th. This again, may be because respondents were willing to purchase DTC genetic tests before, whereof many do not support partial (cost) coverage by insurance (Thiebes et al., 2020).

**Effect (attribute)**	**LogWorth (bar chart)**	**P-Value**
Price***	22.646	0.00000
Reselling of genome data***	11.855	0.00000
No Choice Indicator***	7.499	0.00000
Data processing***	4.244	0.00006
Sampling Site***	3.783	0.00017
Genome test type	1.732	0.01854
Sample storage	1.675	0.02111
Partial coverage by insurance	1.446	0.03581
Consumer research consent	1.218	0.06053
Distribution channel	0.972	0.10665
Business purpose	0.649	0.22419
Additional value sub	0.568	0.27043
Data storage	0.556	0.27794
Test purpose	0.449	0.35540
Region of operation	0.296	0.50581
Sampling kit provider	0.075	0.84071
Data ownership	0.025	0.94308
Note: ** $p \leq 0.01$, *** $p \leq 0.001$		

Table S4-1: Effect summary of choice model for respondents who have taken a DTC genetic test before

Part-Worth Utilities of Attributes

Analysis of marginal utilities per level allows deeper insights into fairness perception difference between the general public and DTC test takers. The marginal utility table (Table S4-2) provides the part-worth utility and marginal probability for every attribute level. Regarding the five significant effects fairness perception of their individual levels is in line with the main analysis (i.e., the same levels are preferred or disliked). However, many of the part-worth utilities are substantially larger. For example, the utility for not selling genetic date increases from 0.28 to 0.47 or lab collection as sampling site from -0.23 to -0.40. Both indicating a greater emphasis on not reselling data ore needing to go to a lab for sample collection.

Regarding the not significant effects, there are some level preference deviations from the main analysis, which could indicate that respondents having taken a DTC genetic test before form a distinct consumer segment, especially considering that some of the insignificances are most likely from the small sample size.

**Attribute**	**Level**	**Marginal Utility**	
		**Utility**	**Probability**
Test purpose	Health test	0.125	0.376
	Lifestyle test	-0.095	0.302
	Relationship test	-0.030	0.322
Business purpose	For profit	-0.080	0.460
	Nonprofit	0.080	0.540
Region of operation	Local	-0.045	0.477
	Worldwide	0.045	0.523
Consumer research consent	Data not used	-0.198	0.270
	Mandatory	0.026	0.338
	Optional	0.172	0.392
Distribution channel	Healthcare professionals only	0.052	0.348
	Internet only	-0.190	0.273
	Multi-contact service	0.137	0.379
Sampling Site	Home collection	0.018	0.384
	Home or Lab collection	0.228	0.403
	Lab collection	-0.407	0.213
Sampling kit provider	Service provider	-0.053	0.316
	Service provider or Third party	0.010	0.336
	Third party	0.043	0.348
Sample storage	Consumer decision	0.175	0.390
	Mandatory	-0.263	0.252
	Never	-0.090	0.358
Genome test type	Genotyping	-0.068	0.306
	Genotyping or Sequencing	0.245	0.419
	Sequencing	-0.177	0.275
Data storage	Database for service provider	0.118	0.373
	Isolated storage	0.012	0.336
	No storage	-0.130	0.291
Data ownership	Consumer	-0.007	0.497
	Service provider	0.007	0.503
Data processing	Basic interpretation	-0.145	0.278
	No interpretation	-0.227	0.256
	Value-added interpretation	0.372	0.466
Additional value subscription	No	-0.073	0.464
	Yes	0.073	0.536
Partial coverage by insurance	No	-0.139	0.431
	Yes	0.139	0.569
Reselling of genome data	No	0.470	0.719
	Yes	-0.470	0.281
Price	For each additional +$1	-0.0015	-
No choice indicator	-	-0.612	-

Table S4-2: Marginal utility and probability for respondents who have taken a DTC genetic test before

The first important deviation occurs for consumer research consent. In contrast to the main analysis, data not used is less desirable than mandatory, with optional still being the preferred choice. While surprising, this may indicate that DTC test takers, would rather see their genetic data used for research mandatorily, than not at all. Another change can be found for *sampling kit provider*, where our results indicate test takers prefer third party providers over service provider, with the option for both once again remaining in the middle. One possible explanation is that many DTC genetic testing service providers accept the use of genetic data, that has been previously collected from a different service provider for a discounted price. As such it makes sense, that respondents, who have already taken a test might want to re-utilize their data, also omitting the need for another sample collection all together. The last major difference occurs for the *data storage* attribute, whereas in the main analysis database for service provider was the least desirable level, for our analysis of DTC test takers it is the most desirable, with a higher utility than isolated or no storage. While in line with respondents preferring mandatory *consumer research consent*, as service providers building a database for their services also utilize the genetic data for research, for example, to improve their tests, it is nonetheless surprising, that many test takers seem to prefer use of their data. Finally, *data ownership* also has swapped levels, where respondents prefer ownership by the service provider. However, because of the low significance the levels also hold minimal utility.

References

de Bekker-Grob, E. W., Donkers, B., Jonker, M. F., & Stolk, E. A. (2015). Sample Size Requirements for Discrete-Choice Experiments in Healthcare: A Practical Guide. *The Patient - Patient-Centered Outcomes Research, 8*(5), 373-384. doi:10.1007/s40271-015-0118-z

Thiebes, S., Toussaint, P. A., Ju, J., Ahn, J.-H., Lyytinen, K., & Sunyaev, A. (2020). Valuable Genomes: Taxonomy and Archetypes of Business Models in Direct-to-Consumer Genetic Testing. *J Med Internet Res, 22*(1), e14890. doi:10.2196/14890
