# Supplementary material for: Perceived fairness of direct-to-consumer genetic testing business models
Source: Electron Mark. 2022 Jul 18;32(3):1621–38. doi: 10.1007/s12525-022-00571-x (PMC9294841; doi:10.1007/s12525-022-00571-x)
Supplement: Supplementary file 5 — (PDF 214 KB) [file 12525_2022_571_MOESM5_ESM.pdf]

Electronic Markets
The International Journal on Networked Business

Perceived Fairness of Direct-to-Consumer Genetic Testing Business Models

Supplementary Material 5

Utility of Direct-to-Consumer Business Models Archetypes

The presented choice model also allows for comparison of existing business models in terms of their perceived fairness through overall utility comparison. Table S5-1 lists the overall utility of 12 service providers examined by Thiebes et al. (2020). These service providers were selected, as they are typical examples of the six archetypes of DTC business models, proposed by Thiebes et al. (2020). To accommodate the changes made to the relevant attributes of DTC business models, a single genetic test was chosen from each service provider, as offered on their websites on May 18th, 2022. This step was necessary to define the *test purpose* and the *price* for the test. For example, 23andMe offers an *Ancestry + Trait Service* (lifestyle test) for $99 and a *Health + Ancestry Service* (health test) for $199. All other data were taken from the original taxonomy (Thiebes et al., 2020).

Service Provider	Archetype	Utility	Test Purpose	Test Name	Price
23andMe	1	-0.720	Lifestyle	Ancestry+Trait Service	$99
AncestryDNA	1	0.302	Lifestyle	AncestryDNA	$99
African Ancestry	2	0.085	Lifestyle	MatriClan Test	$299
FitGenes	2	-0.012	Health	Food Choice	$149
Alpha Biolabs	3	-0.219	Relationship	DNA Paternity Test Kit	$125
DNA Diagnostics Center	3	-1.235	Relationship	Legal Paternity Test	$499
Pillcheck	4	-0.355	Health	Medication Optimization Service	$405
International Biosciences	4	0.134	Health	SkinCareDNA Test	$175
Genetic Genie	5	0.214	Health	GenVue Discovery	$0
Promethease	5	-0.068	Lifestyle	SNPedia Reports	$12
Dante labs	6	-0.295	Health	30X WGS Test	$550
Nebula Genomics	6	-0.635	Health	Deep WGS 30X	$299

Table S5-1: Effect summary of the discrete choice experiment

The calculated utilities indicate that AncestryDNA implements the most desirable business model with a utility of 0.302, while the least desirable business model is adapted by DNA Diagnostics Center (utility of -1.235). Consequently, all inspected business models are well within the boundaries of the hypothetical most (utility of 1.956) and least (utility of -3.266) fairest business models in terms of their utility. It should be highlighted once

again, that the specific test chosen, and its price can have significant impact on the overall utility of the service provider. For example, Nebula Genomics also offers an *Ultra Deep Whole Genome Sequencing 100X* test for $999, which would result in a utility of -1.545 instead of the presented -0.635.

The utility radar charts of Figure S5-1 to Figure S5-12 show the service providers and their respective utility values for every attribute. They allow for visual comparison of the different business models and which attributes they differ in. Within the figures, the utility for each dimension increases from the center outwards of the charts. Interestingly, the examined business models of archetypes two, four, and five seem to be very similar in terms of their attribute specific part-worth utilities, while business models for the archetypes one, three, and six vary more. Further analysis provides some novel insights on the desirability of certain DTC business model archetypes. First, service providers from the first archetype have rather extreme utilities (cf. Figure S5-1 and S5-2) compared to the other investigated business models. In contrast, services located in the second archetype have a comparatively moderate utility (cf. Figure S5-3 and S5-4). While both archetypes offer *Direct-to-Consumer genomics for enthusiasts*, business models from the second archetype differ in the attributes concerning the consumer's data privacy (i.e., the genome data remains property of the consumer, is not stored, is not sold for revenue, and usually not utilized for research). As the results of this DCE indicate, that consumers value these attribute levels higher, it is likely that this causes on average higher desirability. However, tests offered in archetype two also have a higher price than tests from archetype one. Hence, the results indicate that consumers perceive the *high-privacy Direct-to-Consumer genomics for enthusiasts* to be fairer than the first business model archetype. Second, service providers offering relationship tests (found in the third archetype *specific information tests*) also have a rather low utility (cf. Figure S5-5 and S5-6). Analysis of part-worth utility for the *test purpose* also showed that relationship tests are the least desirable in terms of fairness. Third, in contrast to relationship tests, some DTC health tests have a higher utility, proposing a stronger desirability for this kind of test (cf. Figures S5.3, S5-8, and S5-9). However, consumers prefer *simple health tests* as described by archetype four compared to *comprehensive tests with low data processing* (archetype six). While the latter offer potentially more accurate testing results, they often require subsequent interpretation of health care professionals. Last, nonprofit service providers (as prominently found in the *basic low-value Direct-to-Consumer genomics* archetype 5) seem to have a rather high utility (cf. Figure S5-9 and S5-10). However, many services require prior genome sequencing by another service provider (Thiebes et al., 2020). Hence, consumers might refrain from these services as it requires a purchase from other less desirable service providers such as 23andMe.

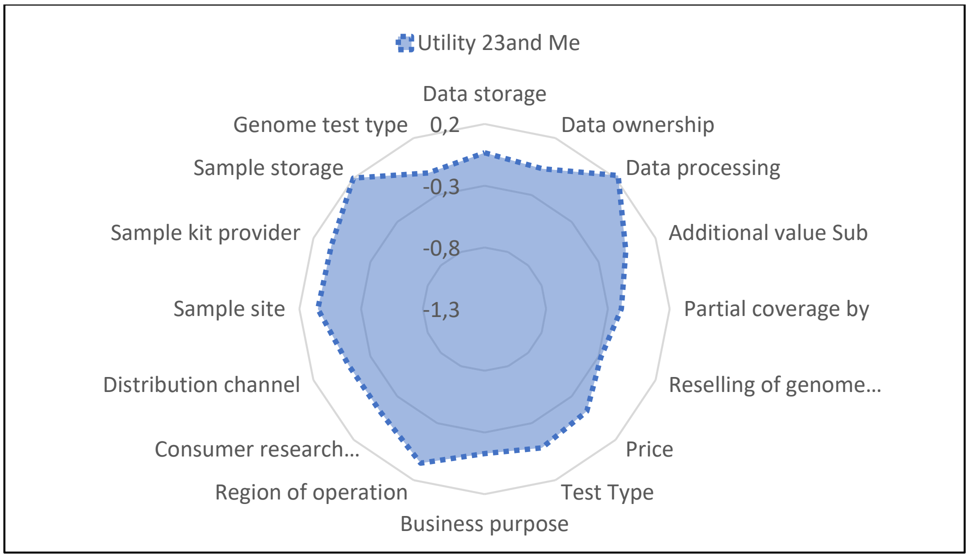

Figure S5-1: Utility chart 23andMe (Archetype 1)

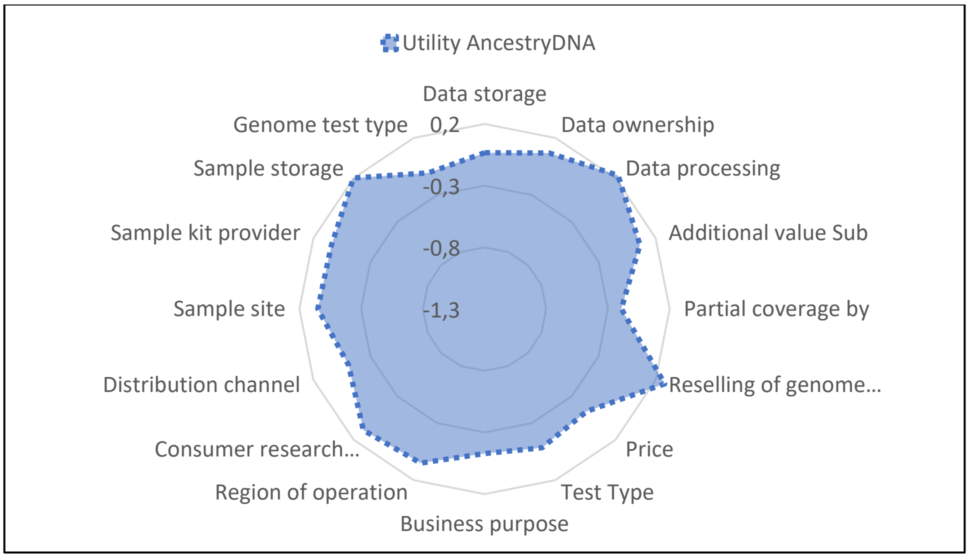

Figure S5-2: Utility char AncestryDNA (Archetype 1)

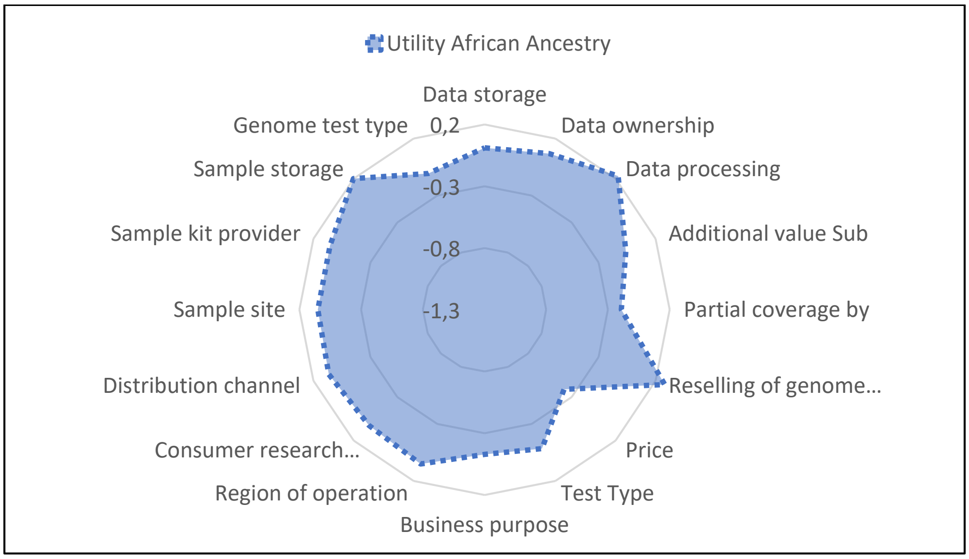

Figure S5-3: Utility chart African Ancestry (Archetype 2)

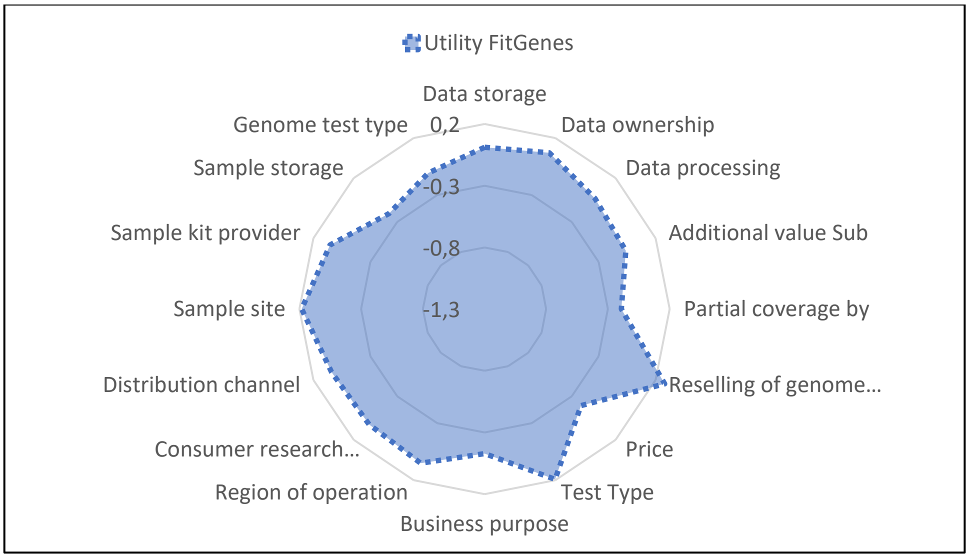

Figure S5-4: Utility chart FitGenes (Archetype 2)

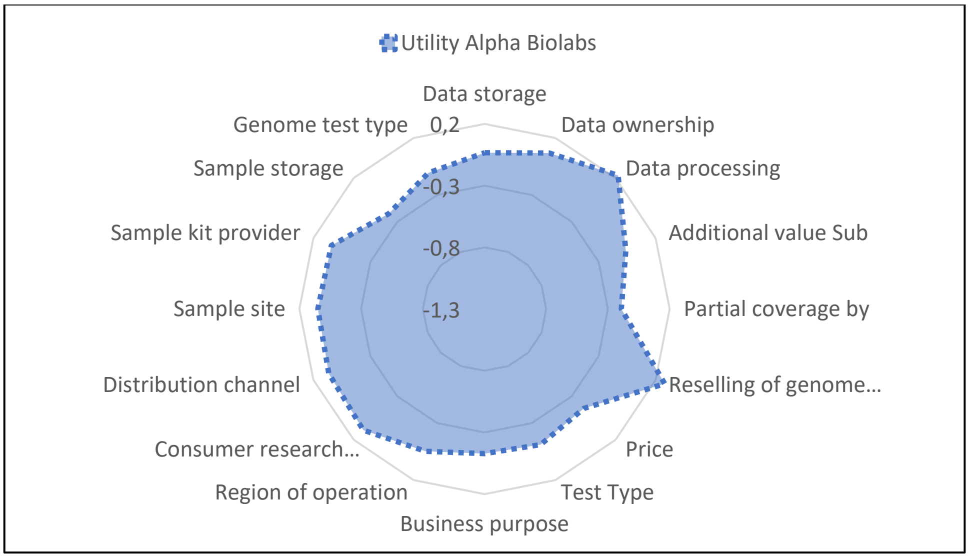

Figure S5-5: Utility chart Alpha Biolabs (Archetype 3)

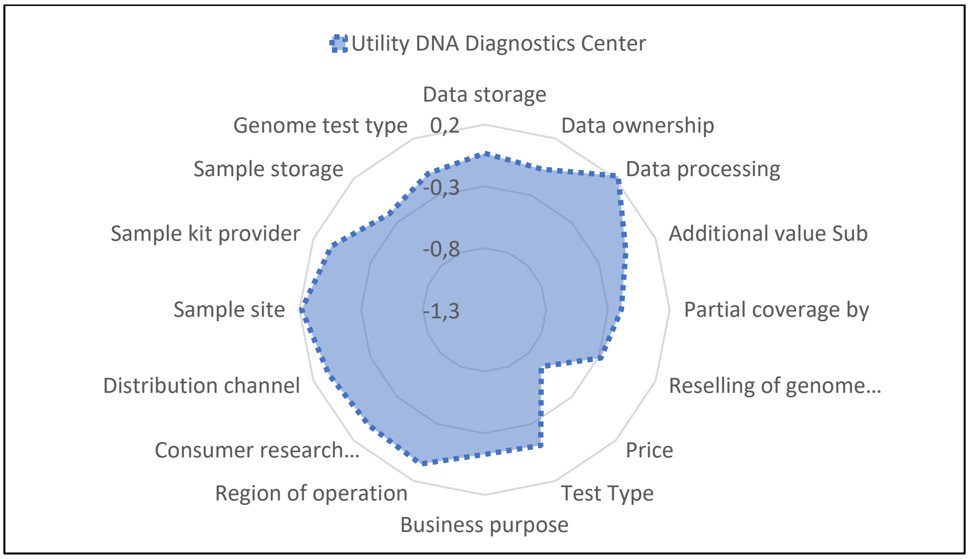

Figure S5-6: Utility chart DNA Diagnostics Center (Archetype 3)

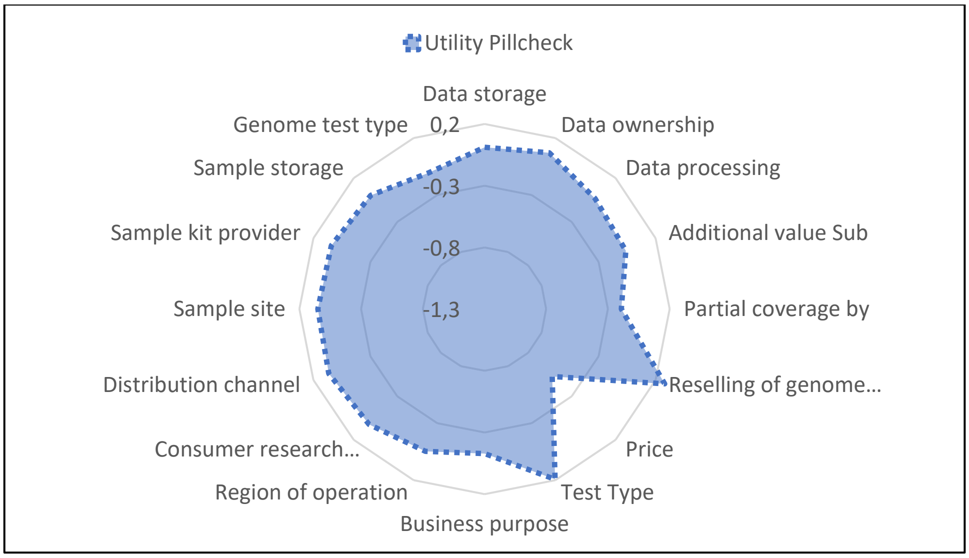

Figure S5-7: Utility chart Pillcheck (Archetype 4)

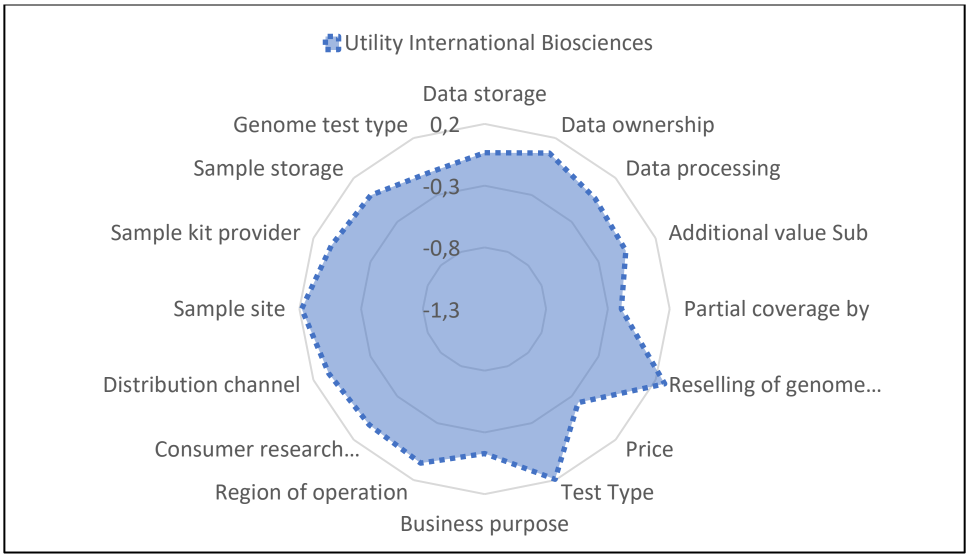

Figure S5-8: Utility chart International Biosciences (Archetype 4)

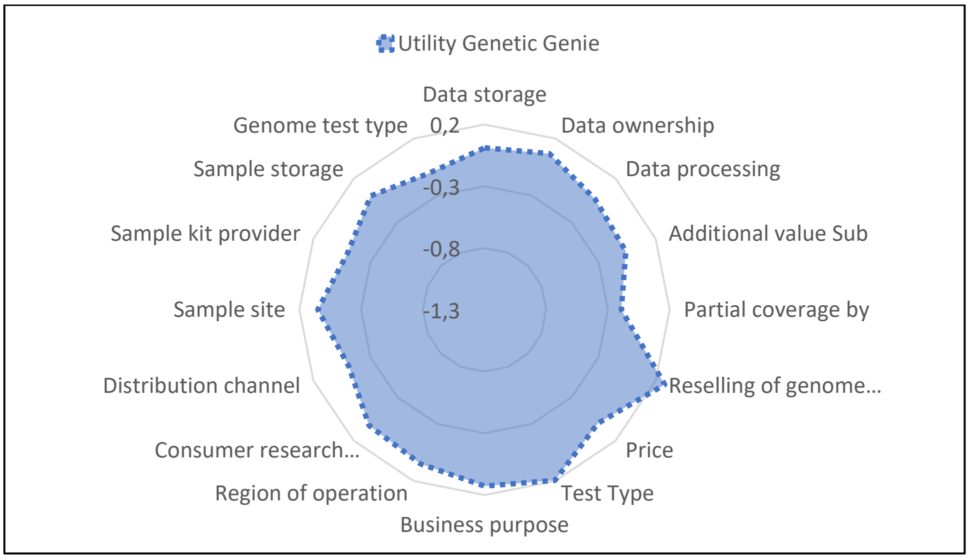

Figure S5-9: Utility chart Genetic Genie (Archetype 5)

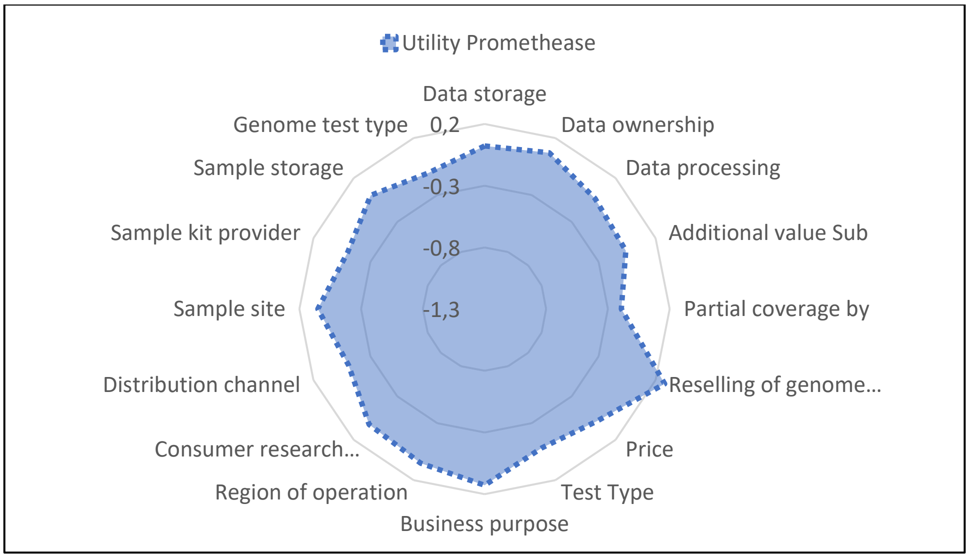

Figure S5-10: Utility chart Promethease (Archetype 5)

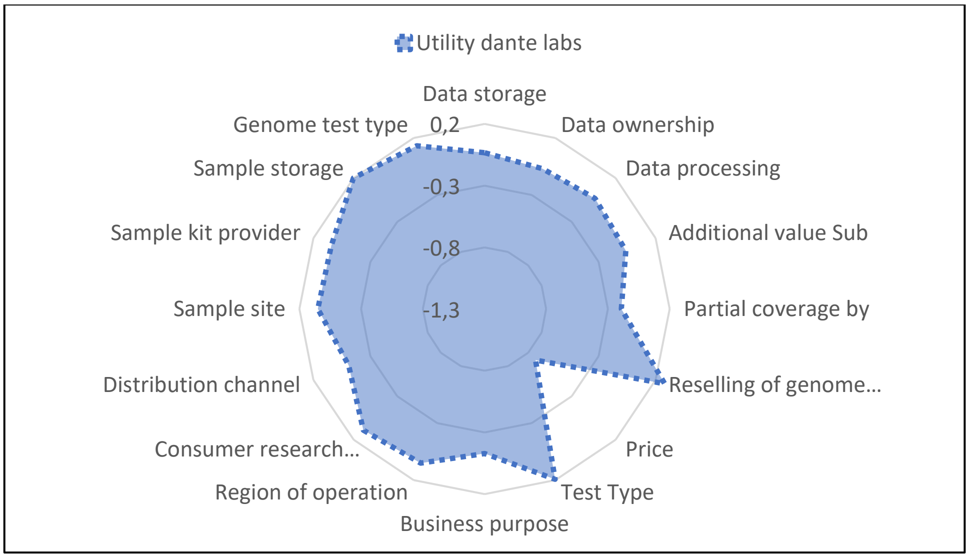

Figure S5-11: Utility chart dante labs (Archetype 6)

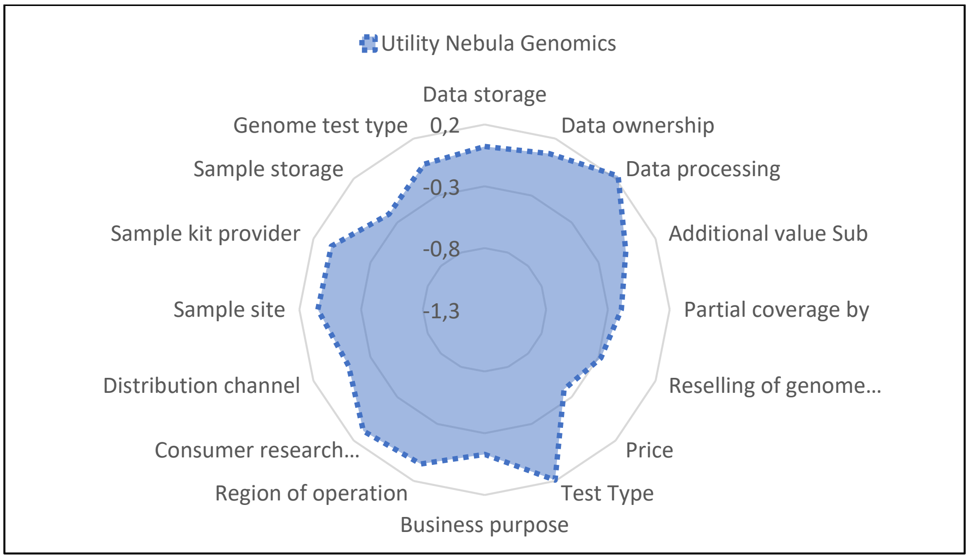

Figure S5-12: Utility chart Nebula Genomics (Archetype 6)

References

Thiebes, S., Toussaint, P. A., Ju, J., Ahn, J.-H., Lyytinen, K., & Sunyaev, A. (2020). Valuable Genomes: Taxonomy and Archetypes of Business Models in Direct-to-Consumer Genetic Testing. *J Med Internet Res, 22*(1), e14890. doi:10.2196/14890
